# A Pilot Randomised Trial of Induced Blood-Stage *Plasmodium falciparum* Infections in Healthy Volunteers for Testing Efficacy of New Antimalarial Drugs

**DOI:** 10.1371/journal.pone.0021914

**Published:** 2011-08-22

**Authors:** James S. McCarthy, Silvana Sekuloski, Paul M. Griffin, Suzanne Elliott, Nanette Douglas, Chris Peatey, Rebecca Rockett, Peter O'Rourke, Louise Marquart, Cornelius Hermsen, Stephan Duparc, Jörg Möhrle, Katharine R. Trenholme, Andrew J. Humberstone

**Affiliations:** 1 Queensland Institute for Medical Research, University of Queensland, Brisbane, Australia; 2 Q-Pharm Pty Ltd, Brisbane, Australia; 3 Queensland Pediatric Infectious Diseases Laboratory, Herston, Australia; 4 Radboud University Nijmegen Medical Centre, Nijmegen, The Netherlands; 5 Medicines for Malaria Venture, Geneva, Switzerland; Menzies School of Health Research, Australia

## Abstract

**Background:**

Critical to the development of new drugs for treatment of malaria is the capacity to safely evaluate their activity in human subjects. The approach that has been most commonly used is testing in subjects with natural malaria infection, a methodology that may expose symptomatic subjects to the risk of ineffective treatment. Here we describe the development and pilot testing of a system to undertake experimental infection using blood stage *Plasmodium falciparum* parasites (BSP). The objectives of the study were to assess the feasibility and safety of induced BSP infection as a method for assessment of efficacy of new drug candidates for the treatment of *P. falciparum* infection.

**Methods and Findings:**

A prospective, unblinded, Phase IIa trial was undertaken in 19 healthy, malaria-naïve, male adult volunteers who were infected with BSP and followed with careful clinical and laboratory observation, including a sensitive, quantitative malaria PCR assay. Volunteers were randomly allocated to treatment with either of two licensed antimalarial drug combinations, artemether–lumefantrine (A/L) or atovaquone-proguanil (A/P). In the first cohort (n = 6) where volunteers received ∼360 BSP, none reached the target parasitemia of 1,000 before the day designated for antimalarial treatment (day 6). In the second and third cohorts, 13 volunteers received 1,800 BSP, with all reaching the target parasitemia before receiving treatment (A/L, n = 6; A/P, n = 7) The study demonstrated safety in the 19 volunteers tested, and a significant difference in the clearance kinetics of parasitemia between the drugs in the 13 evaluable subjects, with mean parasite reduction ratios of 759 for A/L and 17 for A/P (95% CI 120–4786 and 7–40 respectively; p<0.01).

**Conclusions:**

This system offers a flexible and safe approach to testing the *in vivo* activity of novel antimalarials.

**Trial Registration:**

ClinicalTrials.gov NCT01055002

## Introduction

Significant advances have been made recently in the control of malaria [1], [2], with reductions in the incidence and prevalence of infection as well as in malaria-associated mortality. Despite this, half of the world's population remains at risk of infection, with an estimated 225 million cases occurring in 2009, resulting in an estimated 781,000 deaths [2]. While the reduction in burden of malaria is likely to be due to multiple factors, including the increasing use of insecticide-treated bed nets, the replacement of ineffective antimalarials with artemisinin combination therapy (ACT) has been recognized as a major factor in reducing the burden of malaria [2]. However, the most deadly species of malarial parasites, *Plasmodium falciparum*, has successfully developed resistance to almost every antimalarial drug currently marketed. The concerning reports of slow clearance of *P. falciparum* parasitemia following artemisinin therapy in southeast Asia [3] reinforce the requirement to develop new antimalarials to replace drugs losing their efficacy [4]. Additional reasons for the development of new antimalarials include the need to substitute drugs with unfavourable pharmacokinetic or toxicology profiles, and to develop drugs that can be given safely to pregnant women and young children.

Over the last decade there has been a concerted effort to discover and develop new antimalarial agents [5]. At the development end of the pipeline, new fixed-dose artemisinin-based combination therapies (ACTs) are nearing approval, and synthetic alternatives to artemisinins, potentially able to treat emerging resistant strains are in early stages of clinical development (ClinicalTrials.gov Identifier: NCT01213966). On the discovery stage, screening of several million compounds has resulted in the identification of thousands of new leads with antimalarial activity, allowing optimization and selection of new agents with new mechanisms of action [6], [7], [8].

The selection of the most promising new agents for formal development necessitates the assessment of their safety and efficacy via extensive *in vitro* and animal testing, followed by Phase I safety and pharmacokinetic studies in healthy human volunteers, followed by Phase II clinical studies to establish an effective dose and regimen. However, it is not possible to predict with confidence the effective starting doses in patients based on *in vitro* and animal testing. Therefore, the initial Phase II studies in humans must be carefully designed to safeguard the patient in the event of an ineffective dose, while allowing enough time to observe the effect of the agent.

The predominant method for gathering preliminary efficacy data for candidate antimalarials has been to undertake trials in malaria-endemic areas with individuals with patent parasitemia [9], [10]. Such approaches are hindered by a number of factors, including the effect of clinical immunity [11], and the ethical and logistical problems of using a drug of unproven efficacy in subjects with clinical illness who could quickly develop to severe disease. Although trials in asymptomatic, parasitaemic subjects are safer than those in symptomatic patients, the results of such trials in clinically immune subjects may not be predictive of a drug's efficacy in non-immune subjects. Further, the fall in malaria transmission now being widely reported [1], will likely result in an increase in the logistic challenges and expense of undertaking such trials in malaria-endemic areas, particularly with the growing number of anti-malarial drugs in preclinical development. Alternate approaches include testing for clearance of experimental infection, induced either with sporozoites from mosquito bites [12] or by injection of cryopreserved and thawed sporozoites (ClinicalTrials.gov Identifier: NCT01086917) or blood-stage parasites (BSP) within infected erythrocytes.

Induced blood stage infection was widely practiced following the description of malariatherapy for syphilis, for which Julius Wagner-Jauregg won the Nobel Prize in 1927. After the Second World War, experimental studies using blood stage challenge were undertaken for a number of purposes, including testing the efficacy of blood stage schizonticides [13], [14], [15], [16], [17], [18]. Indeed, this approach was utilised in the successful development of halofantrine [19]. These studies were undertaken using a variety of strain of *P. falciparum*, with varying chloroquine sensitivity [18]. However, this approach was largely abandoned in the 1980's due to ethical and safety concerns.

Experimental blood stage infection was revived in the 1990's as a method for testing the efficacy of vaccines acting against the blood stage of malaria infection [20]. Published experience with the stored bank of blood-stage inocula that was prepared for these studies now amounts to 40 malaria-naïve volunteers in six clinical studies [20], [21], [22], [23], [24]. These have demonstrated safety in the volunteers, the reliability of this approach, and a more uniform parasite growth rate after BSP challenge compared to mosquito challenge [23]. Of note the strain of *P. falciparum* used is the chloroquine-sensitive reference strain, 3D7, the same strain whose genome has been sequenced. The safety for volunteers has been further augmented by the availability of artemisinin antimalarials that rapidly clear the parasitemia [25], and by the development of rapid, sensitive and robust real-time PCR assays for quantification of parasitemia [26]. We hypothesized that blood challenge would provide a system for assessing the efficacy of new antimalarial drug candidates for the treatment of *P. falciparum* malaria.

We have undertaken a randomised, unblinded, Phase IIa trial in healthy, malaria-naïve, male adults aiming to assess the feasibility and safety of induced blood stage malaria challenge as a method for assessment of efficacy of new drug candidates for the treatment of *P. falciparum* infection. The study was designed as an enabling study to inform subsequent trial design, and to identify endpoints for assessing new candidate antimalarial drugs in development. Two registered antimalarial drugs were selected as reference treatments, one the relatively fast acting artemisinin combination artemether-lumefantrine, and the other the relatively slow acting drug combination atovaquone-proguanil [25]. Secondary objectives included: i) confirming the parasite growth curves after intravenous (I.V.) inoculation of healthy volunteers with *P. falciparum* blood stage parasites, ii) establishing parasite clearance profiles as measured by PCR after administration of the reference drugs when administered at a target parasitemia of ≥1,000 parasites/mL, and iii) assessing the safety of this system of induced blood stage malaria infection.

## Materials and Methods

### Ethics Statement

The study was approved by the Queensland Institute of Medical Research Human Research Ethics Committee (QIMR-HREC) and reviewed by the MMV Global Safety Board. The study was conducted in accordance with the Declaration of Helsinki principles for the conduct of clinical trials and the International Committee of Harmonization Good Clinical Practice Guidelines as recognized by the Australian Therapeutic Goods Administration (TGA) (www.tga.gov.au/docs/pdf/euguide/ich/ich13595.pdf). The trial was conducted with regulatory oversight by the TGA scheme (CTN # 2010/0042) and registered at the ClinicalTrials.gov (NCT01055002). The protocol for this trial and supporting CONSORT checklist are available as supporting information; see Protocol S1, Checklist S1 and Protocol Amendment S1.

The production of the parasite inoculum has been described previously [20]. In brief, laboratory-reared *Anopheles stephensi* mosquitoes were infected by membrane feeding on a blood meal containing gametocytes with the chloroquine-sensitive *P. falciparum* clone 3D7 derived from an isolate that had collected from an airport worker in Amsterdam. Ten days after their last blood meal, the mosquitoes were allowed to feed on a healthy male who had no evidence of any blood borne virus infection. A 500 mL unit of blood was taken from the volunteer six hours after he became ill with the development of high fever, 13 days after the mosquito bites. The blood was leucocyte depleted, mixed with the cryopreservation agent Glycerolyte 57, aliquoted into 1-mL cryovials and stored in liquid nitrogen at QIMR. Prior to use of the aliquots, the volunteer was repeatedly evaluated for any evidence of seroconversion to blood borne infections in order to eliminate the risk of transferring infection to the recipient volunteers. Up to date, 16 years after collection of the blood the volunteer has remained healthy.

To prepare the inocula, an aliquot of the seed stock was thawed, washed, and the inoculum prepared by diluting to the appropriate dose and volume and dispensed aseptically into 2 mL syringes in preparation for individual volunteer administration. The number of viable parasites in the inoculum was verified in retrospect by quantitative PCR as described below. It was planned that each injected inoculum would contain ∼120,000 erythrocytes, of which ∼5,400 were parasite-infected. As previous experience had indicated that ∼30% of blood stage parasites were viable [20], the final dose was planned to be ∼1,800 viable intraerythrocytic parasites. Each challenge inoculum was dispensed into syringes and stored in sealed plastic bags on ice until administered. The time between thawing and injection was required to be ≤60 minutes.

The inoculum size in terms of parasite genome equivalents and parasitemia from clinical blood samples taken during the trial were quantified by a Real-time Quantitative PCR assay as described elsewhere [26]. In brief, whole blood samples were collected into 2 mL EDTA vacutainers (BD Australia) at 12 hour intervals from Day 3, with additional samples collected at 6 hour intervals in the 48 hours following inoculation. Vacutainers were centrifuged for 5 minutes at 2,500 rpm; 500 µl of packed red cells was removed and added to 500 µl of PBS and mixed. The sample was then divided into two aliquots, one 500 µL aliquot was mixed with 400 µl of Qiagen AL Lysis buffer and DNA extracted using the QIAamp DNA Mini kit following the manufacturer's protocol. A known concentration of equine herpes virus (EHV) was added to each specimen to monitor the efficiency and reliability of the extraction process. Nucleic acids were eluted in 100 µl of elution buffer and stored at −80°C until PCR reactions were performed. The PCR primers and cycling conditions were as previously described [27]. Briefly, the reaction mix consisted of 12.5 µl Quantitect Probe PCR Mix (Qiagen, Australia), 10 pmol of each primer (PerFAL-Forward CTTTTGAGAGGTTTTGTTACTTTGAGTAA and PerFAL-Reverse TATTCCATGCTGTAGTATTCAAACACA), 4 pmol of probe (PerFAL-probe Fam-TGTTCATAACAGACGGGTAGTCATGATTGAGTTCA-BHQ1)and 5 µl of template in a 25 µl final reaction was performed in a Rotorgene 3000 or 6000 (Qiagen, Australia) under the following conditions: 15 min incubation at 95°C, followed by 45 cycles of 95°C for 15 s and 60°C for 1 min. PCR product quantitation was undertaken using a standard curve prepared by serial dilutions of control blood samples with parasitemia as determined by flow cytometry [28]. Six standards were used on each run ranging from 6.4×10^5^ to 6.4 parasites per 500 µL of packed red blood cells (approximating 1 mL of blood at a 50% haematocrit). Two negative controls were included on each PCR run. Parasite concentrations were calculated using the Qiagen, Rotorgene software. Specimens were tested by PCR in duplicate and the average parasite concentration for these two values is reported. The limit of detection of the assay was determined to be 64 parasites per 500 µL of packed red blood cells, approximating a count of 64 per mL, assuming a hematocrit of 45.

Participants were recruited at the trial site, Q-Pharm Pty Ltd, Brisbane, QLD, Australia from January 2010 until June 2010. The volunteers were randomized across the three cohorts 1∶1 to the two registered antimalarials artemether-lumefantrine (Riamet™, Novartis) and atovaquone-proguanil (Malarone™, GSK), both administered according to their approved dosing schedules (4 tablets of Artemether 20 mg/Lumefantrine 120 mg, administered at time 0, 12, 24, 36, 48, and 60 hours; 4 tablets of Atovaquone 250 mg/Proguanil100 mg administered at 0, 24, 48 hours). Randomization was undertaken by the study pharmacist using the SPSS software package to generate a treatment allocation document. As this was a pilot trial and the principal outcome variables to be analysed were the PCR-determined parasite clearance kinetics, both the volunteers and the investigators were unblinded. All PCR-based laboratory testing of the participant blood samples was undertaken in a blinded fashion. It was required that the interval between successive cohorts be at least 13 days to enable review by Safety Review Team at least 10 days following completion of treatment in the previous cohort. Following screening and informed consent, healthy, malaria naive male volunteers were intravenously inoculated on Day 0 with ∼5,400 *P. falciparum*-infected human erythrocytes. On an outpatient basis, volunteers were monitored each morning and evening from Day 3 to Day 7 after inoculation for adverse events and the unexpected early onset of symptoms, signs or parasitological evidence of malaria. If clinical or parasitological evidence of malaria (either the identification of two or more malaria parasites on a malaria thick film, platelet count less than 100×10^9^/L, or the onset of clinical features of malaria) or parasitemia of ≥1,000 parasites/mL as determined by PCR was detected before the evening of Day 6, the protocol required that allocated treatment begin at this time. On the evening of Day 6, volunteers were to be admitted to the study unit and confined for safety monitoring and antimalarial treatment. For the second and third cohorts, continuation of outpatient monitoring was provided, if the growth rate of the malaria parasites as determined by PCR was slower than expected. For cohort one, antimalarial treatment was designated to begin on the evening of Day 6 irrespective of parasite count. For cohorts two and three, the timing of administration of antimalarial therapy was modified such that it was planned to begin on the evening after the first recording of a parasitemia ≥1,000 parasites/mL. Following treatment, volunteers were followed as inpatients for at least 36 hours, (2 evenings) to ensure tolerance of the therapy and clinical response, then, if they were clinically well, they were on an outpatient basis for safety and continued presence of malaria parasites via PCR and thick blood film review. Adverse events were monitored either via telephone, or within the clinical research unit and on outpatient review after inoculation and antimalarial study drug administration.

A total of 36 healthy, malaria naïve male volunteers, aged between 18 to 45 years were recruited from a database of healthy volunteers maintained by Q-Pharm, or by advertisement to students of Queensland universities and the general community. Screening medical histories and physical examinations were performed after written informed consent had been obtained. Details of the inclusion and exclusion criteria are available in Protocol S1.

Vital signs (temperature, heart rate, blood pressure and respiratory rate) were measured on Day 0 then at least daily from Days 3–13 and blood was collected for safety purposes was undertaken on a regular basis. Volunteers were monitored by a medical investigator during the confinement period in the morning and evening, and at the outpatients visit on Day 0, Days 3–7, Days 9–12, and at the follow up Day 28 visit. An experienced nurse was in attendance at the study centre throughout the period of inpatient confinement. For the duration of the study, a medical Investigator was available within 30 minutes if required. Progress of the trial and review of its safe conduct were overseen by a safety review team including an independent medical expert.

Initial estimates of the required sample size were based on an unpublished dataset of parasite clearance kinetics from a study undertaken at The Radboud University Nijmegen Medical Centre where volunteers had been treated with chloroquine. The group size required to detect a 25% differences in parasite clearance rate between two treatment arms was calculated to be 10 per treatment group for a two sided test at the 5% level of significance with 80% power. After the second cohort was completed, a second sample size calculation was undertaken, and it was determined that a total study size of 14 with seven volunteers in each arm would result in the desired statistical power. The statistical analysis plan entailed comparison of the two groups of drug treated volunteers using analysis of variance of the gradients estimated from the modelling of parasite reduction rates [25]. A cohort effect was tested to look for differences between cohorts, but was found to be unimportant. Analysis entailed testing the effect size along with its 95% confidence interval. The safety analysis plan entailed examining the overall number and percentage of volunteers with at least one AE or SAE in each cohort and over the entire study and included the incidence, intensity, and relatedness to malaria infection or antimalarial drug administered. Parasite clearance curves were analysed by calculating a slope of log transformed parasitaemia against time since treatment with antimalarials using linear regression. The corresponding parasite reduction ratios (PRR) were calculated from the mean slopes of the clearance curves for the two treatment groups. This parameter reflects the fractional reduction in parasitemia per asexual life cycle of 48 hours, and is analagous to the killing rate induced by a specific drug [25]. A one way ANOVA was used to compare the slopes between the different treatments.

## Results

**Figure 1:**
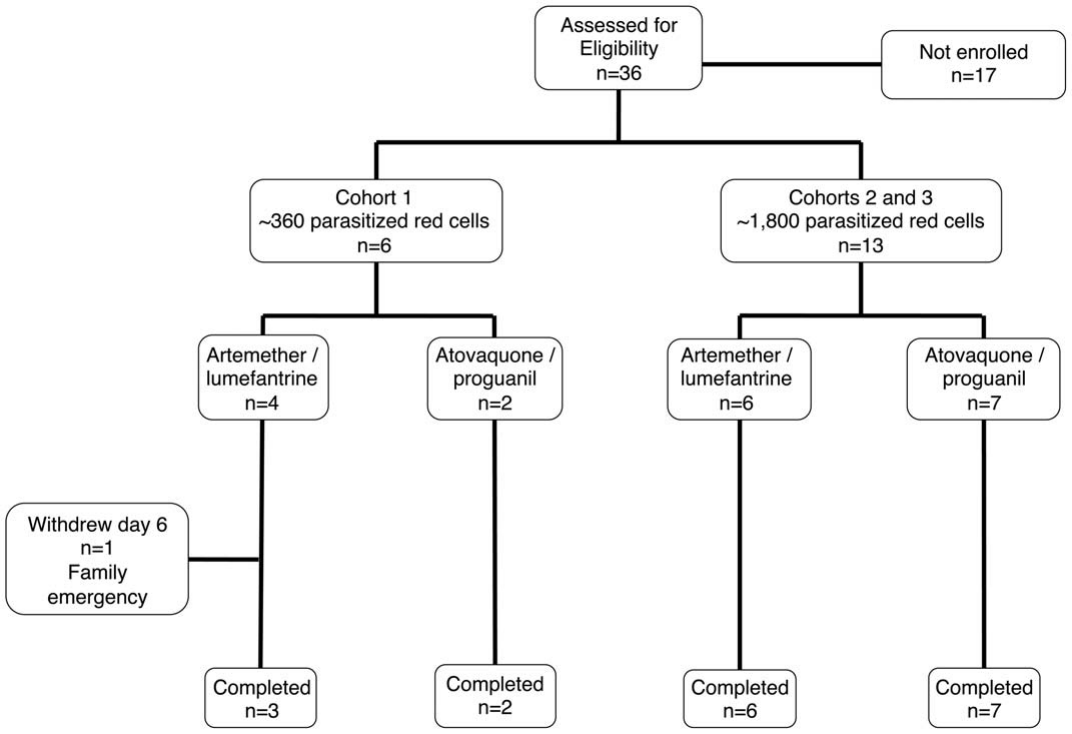
Participant flow.

Of 36 males screened as potential study volunteers, 19 were deemed eligible and enrolled in three sequential cohorts (Figure 1). Reasons for exclusion included the following: declined to participate (n = 6), equivocal HIV antibody test (n = 1), elevated Body Mass Index (n = 1), hemoglobin below 120 g/L (n = 1), needle phobia (n = 1) and positive urine drug screen (n = 1); six eligible volunteers were not required. The median age was 23 years (range 18–42 years); 17 of the enrolled volunteers were of Caucasian background; two were Asian. The study was initially designed to be conducted in two cohorts (n = 6 and n = 10). A miscalculation in preparation of the inoculum in cohort one resulted in the volunteers receiving a dose of parasites 5 fold lower than stipulated. Although all became PCR positive, none reached the target parasitemia of >1,000 parasites/mL before the timepoint when antimalarial treatment was designated to commence (Table S1). Following completion of the first cohort, and safety review a number of changes were made to the protocol. .The second cohort was reduced in size (n = 4); following successful completion of study objectives in this, cohort, a third cohort was enrolled with a planned size of ten volunteers. One subject in the third cohort had a positive urine drug screen on the designated day of inoculation and therefore was removed from the study before receiving the inoculum. Thus the third cohort consisted of nine volunteers.

No serious adverse events were recorded during the study. In the first cohort of 6 volunteers who receivedt ∼360 BSP, no clinically significant symptoms or signs related to the inoculum or drug treatments were noted (Table 1). One volunteer developed transient neutrophilia (13.7×10^9^/L) on Day 9 (Table 2). This resolved spontaneously. One volunteer in cohort one who was randomised to artemether-lumefantrine withdrew from the study on Day 5 due to a family emergency. He completed his antimalarial treatment and returned to complete safety follow-up on Day 28.

**Table 1:** Symptoms reported in the study volunteers.

	Cohort 1 (n = 6)		Cohorts 2 and 3 (n = 13)	
	Artemether/lumefantrine	Atovaquone/proguanil	Artemether/lumefantrine	Atovaquone/proguanil
Malaise			1	1
Chills			1	2
Fever			2	
Sweating			3	
Facial flushing			1	
Myalgia			2	2
Headache			2	4
Neck ache			1	
Back pain				1
Nausea				1
Abdominal discomfort				1
Diarrhoea		1		
Vivid dreams	1			1
Tongue ulcer				1

**Table 2:** Abnormal laboratory values.

	Cohort 1 (n = 6)		Cohorts 2 and 3 (n = 13)	
	Artemether/lumefantrine	Atovaquone/proguanil	Artemether/lumefantrine	Atovaquone/proguanil
Neutrophilia†		1		1
Neutropenia††			4	6
Lymphocytopenia§			2	2
Thrombocytopenia§§			1	
Elevated ALT¶			1	1

† (>11.0×10^9^/L).

†† (<2.0×10^9^/L).

§ (<0.8×10^9^/L).

§§ (<100×10^9^/L; nadir 69×10^9^/L on Day 11).

¶ (>2× normal; <45 IU/mL).

All of the volunteers in cohorts two and three who received a higher dose of parasites of about 1,800 BSP, reached the target parasitemia of ≥1,000 parasites/mL (see below). Symptoms consistent with the early clinical features of malaria were commonly observed (Table 1), with malaise, chills fever headache, neck ache, mylagia and back ache being commonly reported. These symptoms began to appear at peak parasitemia (from Day 7 onwards), were all of only mild to moderate severity, were of two days duration or less, and resolved spontaneously, or after the administration of paracetamol. One volunteer experienced fever to over 39°C with rigors immediately prior to and just after the commencement of antimalarial therapy. Two volunteers required an additional 24 hours of observation beyond the protocol-mandated 36 hours due to persisting low grade fever. The expected mild disturbances in haematologic and biochemical parameters were observed. Moderate neutropenia (<2.0×10^9^/L), and lymphocytopenia (<0.8×10^9^/L) were commonly observed (n = 10 and n = 4 respectively; Table 2). These all occurred at the time of peak parasitemia or soon after the commencement of antimalarial chemotherapy. One volunteer developed transient thrombocytopenia of three days duration, with a nadir platelet count of 69×10^9^/L on Day 11. Transient rises in liver transaminase levels above two times the upper limit of normal occurred in two individuals (ALT 119 U/L on Day 14 in one volunteer, and 117 U/L on Day 17 in the other); both had normalised when retested on Day 28. One volunteer in cohort three was found to have a mild idiopathic neutropenia that was judged unrelated to the study. His screening haematology tests were all in the normal range, but his neutrophil count on Day 0, prior to inoculation was found on retrospect to be 1.60×10^9^/L. His neutrophil count then rose into the normal range before falling below 2.0×10^9^/L on Day 8. His neutrophil count remained below 2.0×10^9^/L until Day 44 when it was 1.64×10^9^/L. Although he did not give a history suggestive of increased frequency or severity of infections or other symptoms, he was referred for specialist assessment by a hematologist, but his hematologic parameters had returned to within the normal range by this time.

**Figure 2:**
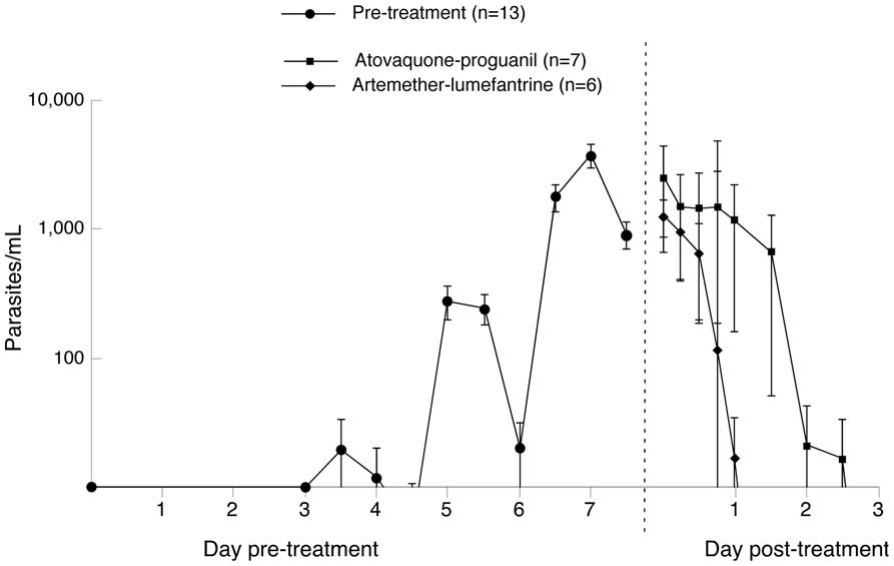
Growth and clearance of parasitemia in study subjects. The mean number of *P. falciparum* parasites before treatment (closed circles; n = 13), and after treatment with artemether-lumefantrine (closes squares; n = 6) or with atovaquone-proguanil (closed diamonds; n = 7), as determined by real-time polymerase-chain-reaction assay. The I bars denote standard errors.

Thick blood films remained negative throughout the study for all volunteers. Figure 2 shows the parasite growth and clearance patterns for the 13 volunteers in cohorts 2 and 3, all of whom reached the designated target parasitemia of >1,000 parasites/mL. The median peak, pre-treatment parasitemia among these volunteers was 2,926 parasites/mL, (range: 1,501–8,524). In the artemether-lumefantrine treatment group parasites were cleared between 1–2 days whereas in the atovaquone-proguanil treatment group parasite clearance was 2.25–3 days. The clearance rates in the artemether-lumefantrine and the atovaquone-proguanil treatments groups were significantly different (p<0.01). The corresponding parasite reduction ratios (PRR), calculated from the mean slopes for the two treatment group were 759 (95% CI 120–4786) and 17 (95% CI 7–40) respectively.

## Discussion

This study has demonstrated the feasibility and safety of induced blood stage malaria infection as a method for assessment of efficacy of antimalarials. When combined with a sensitive, quantitative PCR assay for measurement of parasite growth and clearance it is possible to gain robust clearance kinetic data from a relatively small number of volunteers with submicroscopic level infection. This all occurs largely before the volunteers develop symptoms of malaria. Those volunteers who did develop early clinical symptoms of malaria reported a rapid resolution of symptoms within 48 hours of commencement of antimalarial chemotherapy. The clinical and laboratory abnormalities observed were mild, transient, and not different from those reported in previous human volunteer studies where experimental malaria infection has been undertaken, either by mosquito inoculation [12], [29], [30], [31], [32], which now number over 1,000 volunteers studied, or by blood stage inoculation [20], [21], [22], [23], [24].

The results of the analysis of the kinetics of parasite clearance are in accordance with previous studies demonstrating the more rapid activity of drugs of the artemisinin class over the relatively slow-acting combination of atovaquone-proguanil. These results indicate that this methodology provides an opportunity for assessing the relative speed of clearance of parasites of new agents compared with antimalarial agents with known activity.

While the confidence intervals of the clearance curves were relatively wide in this study in which the cohort sizes were small (six and seven, respectively), the shape of the curves following administration of the antimalarials suggests that parasite clearance begins immediately after administration of the artemisinin drug, while there is a lag with the atovaquone-proguanil combination (Figure 2), an observation in accordance with the hypothesis that the artemisinins act throughout the parasite lifecycle, while the other drug combination acts predominantly late in the parasite lifecycle. It remains to be determined how the clearance kinetics as determined by the sensitive PCR methodology observed at submicroscopic parasitemia in largely asymptomatic volunteers compare to those observed in symptomatic patients with clinical malaria and significantly higher levels of parasitemia.

In many previous studies of the therapeutic efficacy of antimalarial drugs, the clearance of parasitemia has been reported as the parasite clearance time (PCT). This parameter is subject to a number of confounding effects, including the height of parasitemia at enrollment, the background immunity in the population [11], the frequency of collection of blood films, and the expertise and rigor in identifying parasites at low levels. Indeed, in most studies, the threshold for detection of parasitemia in the study has not been reported. A parameter that has been suggested as a more appropriate measure of parasite clearance is the parasite reduction ratio (PRR) [25]. This is the fractional reduction in parasitemia over the parasite lifecycle (48 hours for *P. falciparum*). The PRR values derived in our study were 759 for artemether-lumefantrine and 17 for atovaquone-proguanil. As this value has not been reported in most previous studies of these drug combinations, we undertook a review of previous studies we were able to identify where the starting parasitemia and parasite clearance times were reported. We identified six studies where artemether-lumefantrine was studied [33], [34], [35], [36], [37], [38] and seven for atovaquone-proguanil [39], [40], [41], [42], [43], [44], [45]. To calculate a PRR we assumed that the threshold of detection of parasites on a thick film was 20 parasites per microlitre. In the six studies where sufficient data for artemether lumefantrine were available, the PRR ranged from 217–39,705, while for the seven studies where corresponding data for atovaquone-proguanil were available the PRR ranged from 24–1,620 (Table S2). Thus the PRR value we observed with artemether-lumefantrine is within the range of previously reported values, while that for atovaquone/proguanil it is slightly below the lowest. While there are a number of important differences between these field studies and the experimental system we used, including importantly the level of parasitemia, method of quantification of parasitemia (blood film versus PCR) and the background immunity of the study volunteers, the values derived in this study are within the range reported in these studies. Nevertheless, it will be useful to formally compare the results obtained in this study with the situation that occurs in clinical malaria by undertaking PCR-based study of parasite clearance kinetics following treatment of symptomatic cases.

Recently, there has been significantly more published experience with experimental malaria infections induced by mosquito bite, for studies of immunity and vaccine testing [29], [30], [31], [32] and drug development [12], than by BSP challenge [20], [21], [22], [23], [24]. However, providing that the objective is to focus exclusively on blood-stage effects, experimental infection with BSP offers some significant advantages over infection by sporozoites. These include the ability to tightly control the dose of parasites administered, compared to mosquito inoculation where there is substantial variability in the number of sporozoites inoculated [46], [47]. Earlier studies indicated that after the bite of 5 infected mosquitoes under standard experimental conditions each mosquito injects ∼5–10 sporozoites per bite [46]. However more recent studies indicate that between 100 and 300 sporozoites are inoculated with each bite [47], [48]. As approximately 30,000 merozoites are released into the bloodstream when a single infected hepatocyte ruptures, the starting parasitemia is much lower and more tightly controlled in our BSP experimental system. This provides an added margin of safety and a longer opportunity to observe growth of parasitemia using a sensitive PCR assay. A disadvantage of experimental infection with BSP is the need to inoculate a small volume of donor human blood. This risk has been minimized by exhaustive testing of the donor, the fact that the donor remains healthy over ten years after donating the malaria-infected blood unit, and by the experience to date using this source material without any infectious complications in the recipients. Further, no evidence has accrued of alloimmunization to red cell antigens among recipients of this material (data not shown). However, if the experimental objective is to measure clearance of blood stage parasites, the means of reaching the pre-treatment target parasitemia (by inoculation of sporozoites or blood stage parasites) may be of secondary importance.

Advantages of this system from a drug development perspective include the ability to study an experimental antimalarial agent of unknown efficacy under tightly controlled and monitored conditions, and consequently the opportunity to easily and simultaneously collect rich pharmacokinetic data in order to understand the pharmacokinetic-pharmacodynamic relationship. The reference *P. falciparum* strain used in this study is the best studied strain of *P. falciparum*, with a well characterized drug sensitivity profile. Overall, this provides a margin of safety above that would not pertain if patients with symptomatic malaria were studied in field settings. Thus this system would facilitate the down-selection of ineffective new agents.

The falling prevalence of malaria in many parts of the world [1], weak infrastructure and processes for ethical and regulatory review resulting in unpredictable timelines, and the potentially large number of novel antimalarials that may reach early efficacy evaluation [6], [7], [8] pose specific problems for antimalarial drug development. In this respect the availability of a new clinical capacity to test experimental of antimalarials represents a promising approach to screening novel agents and combinations. The utility of this approach will be better defined once further experience is gained with this system and its predictive in symptomatic patients is defined.

## Supporting Information

Table S1 **PCR-derived parasite counts in study subjects.** Parasite counts at time of treatment commencement are underlined. ND = not detected. Samples that yielded positive PCR results with parasite counts below 64/mL provided qualitative information only and were censored for the purpose of calculating parasite clearance kinetics.
(DOCX)

Table S2 **Parasite Reduction Ratios (PRR) in previous studies of artemether – lumefantrine and atovaquone -proguanil, where the starting parasitemia and parasite clearance times were reported.** To calculate the PRR it was assumed that the threshold of detection of parasites on a thick film was 20 parasites per microlitre.
(DOCX)

Checklist S1 **CONSORT checklist.**
(DOC)

Protocol S1 **Trial protocol.**
(PDF)

Protocol Amendment S1 **Amended trial protocol.**
(PDF)
