# Supplementary material for: A Pilot Randomised Trial of Induced Blood-Stage Plasmodium falciparum Infections in Healthy Volunteers for Testing Efficacy of New Antimalarial Drugs
Source: PLoS One. 2011 Aug 22;6(8):e21914. doi: 10.1371/journal.pone.0021914 (PMC3159571; doi:10.1371/journal.pone.0021914)
Supplement: Table S2 — Parasite Reduction Ratios (PRR) in previous studies of artemether – lumefantrine and atovaquone -proguanil, where the starting parasitemia and parasite clearance times were reported. To calculate the PRR it was assumed that the threshold of detection of parasites on a thick film was 20 parasites per microlitre. (DOCX) [file pone.0021914.s002.docx]

Supplementary Table S2

| Drug | n | Peak parasitemia | PCT (hrs) | PRR | Notes | Reference |
| --- | --- | --- | --- | --- | --- | --- |
| Artemether/  Lumefantrine | 102 | 14,973 | 30 | 39,705 | Hainan | [[33](#_ENREF_33)] |
| Artemether/  Lumefantrine | 309 | 4,336 | 48 | 217 | Thailand | [[34](#_ENREF_34)] |
| Artemether/  Lumefantrine | 120 | 55,017 | 48 | 2,751 | Tanzanian children | [[36](#_ENREF_36)] |
| Artemether/  Lumefantrine | 260 | 21,110 | 32 | 34,292 | Thailand | [[37](#_ENREF_37)] |
| Artemether/  Lumefantrine | 126 | 15,825 | 43 | 1,719 | Thailand | [[38](#_ENREF_38)] |
| Artemether/  Lumefantrine | 359 | 9,889 | 44 | 869 | Thai Adults with MDR Pf | [[35](#_ENREF_35)] |
| Atovaquone/  Proguanil | 71 | 5,030 | 83.3 | 24 | Gabonese Adults | [[39](#_ENREF_39)] |
| Atovaquone/  Proguanil | 77 | 12,059 | 57.5 | 209 | Brazilian Adults | [[40](#_ENREF_40)] |
| Atovaquone/  Proguanil | 32 | 27,776 | 47 | 1,620 | Thai children with MDR Pf | [[41](#_ENREF_41)] |
| Atovaquone/  Proguanil | 82 | 14,799 | 72.0 | 82 | Zambian Adults | [[43](#_ENREF_43)] |
| Atovaquone/  Proguanil | 79 | 38,270 | 65 | 261 | Thailand | [[42](#_ENREF_42)] |
| Atovaquone/  Proguanil | 21 | 21,204 | 60.1 | 261 | Non-immune European adults | [[44](#_ENREF_44)] |
| Atovaquone/  Proguanil | 22 | 3,320 | 72 | 46 | Non-immune Javanese transmigrants to Papua | [[45](#_ENREF_45)] |
