# Supplementary material for: A Pilot Randomised Trial of Induced Blood-Stage Plasmodium falciparum Infections in Healthy Volunteers for Testing Efficacy of New Antimalarial Drugs
Source: PLoS One. 2011 Aug 22;6(8):e21914. doi: 10.1371/journal.pone.0021914 (PMC3159571; doi:10.1371/journal.pone.0021914)
Supplement: Protocol S1 — Trial protocol. (PDF) [file pone.0021914.s004.pdf]

QP09C08: Version 3, 15March2010

A PILOT PHASE II STUDY OF THE EFFICACY OF ANTIMALARIAL DRUGS AGAINST *PLASMODIUM FALCIPARUM* BY EXPERIMENTAL CHALLENGE WITH A LOW DOSE OF BLOOD STAGE PARASITES IN HEALTHY MALE VOLUNTEERS

MEDICINES FOR MALARIA VENTURE
(MMV)

CONFIDENTIALITY STATEMENT

The information contained in this document, is the property of MMV and is therefore provided to you in confidence for review. It is understood that this information will not be disclosed to others without written authorization from MMV

Protocol Number:	**QP09C08**
Title of Protocol:	**A PILOT PHASE II STUDY OF THE EFFICACY OF ANTIMALARIAL DRUGS AGAINST *PLASMODIUM FALCIPARUM* BY EXPERIMENTAL CHALLENGE WITH A LOW DOSE OF BLOOD STAGE PARASITES IN HEALTHY MALE VOLUNTEERS**
Contract Research Organisation:	Q-Pharm Pty Limited<br>Level D, 300C Herston Road,<br>Herston, QLD 4006
Clinical Study Centre:	Q-Pharm Clinics,<br>Level D, 300C Herston Road<br>Level 6, Block 8, Royal Brisbane and Women's Hospital<br>Herston QLD 4006
Principal Investigator:	James McCarthy MBBS
The Principal Investigator is employed by and located at:	**Principal Investigator:**<br>Queensland Institute of Medical Research<br>acting as Visiting Principal Investigator - Q-Pharm Pty Limited<br>Level D, 300C Herston Road<br>Herston QLD 4006
Institutional Ethics Committee to which Q-Pharm is responsible:	The Queensland Institute of Medical Research Human Research Ethics Committee (QIMR-HREC), Herston, Brisbane, Qld
Clinical Laboratory	Pathology Queensland<br>Level 3, Block 7<br>Royal Brisbane and Women's Hospital<br>Brisbane QLD 4029
Statistician	Dr Peter O'Rourke

EMERGENCY CONTACTS

Name	Title/Designation	Location	Telephone/Fax No.	
Dr J. McCarthy	Principal Investigator	QIMR/Q-Pharm	Work	07 3845 3647
		J.McCarthy@uq.edu.au	AH	041 442 4659
Dr. Paul Griffin	Sub-Investigator	RBWH	Work	07 36368111
		Paul_Griffin@health.qld.gov.au	AH	040 207 7302
L M Kear	Clinic Manager	Q-Pharm	Work	07 3845 3622
			AH	0400 803 005
T Starr	Nurse Supervisor	Q-Pharm	Work	07 3845 3632
				0417 628 099
Natasha Hayes	Sponsor's Monitor	Trident Clinical Research	Work	07 32177711
			Fax	07 3217 7511
Dr Stephan Duparc	Medical Director	Medicines For Malaria Venture	Work	+41 79 446 2956
			Fax	+41 22 799 4061

IN THE CASE OF AN EMERGENCY: SERIOUS ADVERSE EVENTS WILL BE REPORTED BY THE PRINCIPAL INVESTIGATOR TO THE SPONSOR WITHIN 24 HOURS.

1. SYNOPSIS

Name of Sponsor/Company: Medicines for Malaria Venture

Name of Investigational Product: Blood stage *Plasmodium falciparum* Challenge Inoculum (BSPC)

Name of Reference Product: artemether/lumefantrine (**Riamet®)and** atovaquone/proguanil hydrochloride (**Malarone®)** **Name of Active Ingredients:**

Riamet®**:** Artemether (20mg) and Lumefantrine (120mg)

Malarone®: Atovaquone (250mg) and Proguanil hydrochloride (100mg)

Title of Study: A Pilot Phase II study of the efficacy of antimalarial drugs against *Plasmodium falciparum* by experimental challenge with a low dose of blood stage parasites in healthy male volunteers

Study center(s): Q-Pharm Pty Limited, Herston, QLD, Australia

Principal Investigator: James McCarthy

Investigators: Paul Griffin, Jo Marjason, Frances Jenkins, Ming Lin, and Indika Leelasena

**Studied period:**<br>Estimated date first patient enrolled: February 2010<br>Estimated date last patient completed: June 2010	**Phase of development:** Phase 2

Objectives:

Primary:

- To validate a human malaria challenge study design and assess appropriate endpoints for assessment of efficacy of new antimalarial drug candidates for the treatment of *Plasmodium falciparum.*

Secondary:

- To confirm the parasite growth curves after I.V. inoculation of healthy volunteers with *P. falciparum* blood stage parasites
- To establish the parasite clearance profiles by PCR after administration of fast and slow acting antimalarial drugs at a target parasitemia of ≥1,000 parasites/ml after inoculation with an experimental malaria challenge
- To assess the safety of an experimental malaria challenge

Methodology: This is a single-center, controlled, randomized, study using a BSP inoculum challenge as a model to assess the activity of antimalarial agents. The study will be conducted in 3 cohorts (n = 6, n= 4 and n = 10). Cohorts 2 and 3 will not commence until at least after day 12 of the previous cohort and review by Safety Review Team following day 9 exit of the previous cohort. The participants will be randomized 1:1 to the two registered antimalarials. This is an enabling study using registered antimalarial drugs as reference treatments (one slow acting and one fast acting), aimed to inform trial design, endpoints and testing regimens for assessing new candidate antimalarial drugs in development.

Cohort 1 had approximately 50 parasites based on retrospective analysis of the innoculum and the growth curves in the volunteers. Following Cohort 1, the dose is to be adjusted, The study will follow the sequence of the challenge inoculation, reference treatment and follow-up. Healthy male participants will be inoculated on Day 1 with ~5300 *Plasmodium falciparum*-infected human erythrocytes administered intravenously. On an outpatient basis, participants will be monitored morning (AM) and night (PM) from day 3 to day 5 for adverse events and the unexpected early onset of symptoms, signs or parasitological evidence of malaria. On day 5 evening, participants will be admitted to the study unit and confined for safety monitoring and antimalarial treatment unless the PCR for malaria parasites is negative on the morning of day 5 when

admission will be deferred until PCR is positive. The participants will be closely monitored and as soon as the PCR quantification is confirmed to be ≥ 1,000 parasites/mL the participants will be administered antimalarial treatment. Reference treatment administration will continue on Day 7 and 8 (3 days of treatment). If clinical or parasitologic evidence of malaria (either the identification of two or more malaria parasites on a malaria thick film, platelet count less than 100 $x10^9$/L, or the onset of clinical features of malaria) occurs or PCR quantification of ≥1,000 parasites/mL is detected before day 6 evening, allocated treatment will begin at this time.

Following treatment, participants will be followed up as inpatients for at least 36 hours, (2 evenings) to ensure tolerance of the therapy and clinical response, then if clinically well on an outpatient basis for safety and continued presence of malaria parasites via PCR and thick blood film review.

Adverse events will be monitored via telephone monitoring, within the clinical research unit and on outpatient review after malaria challenge inoculation and antimalarial study drug administration. Blood samples for safety evaluation, malaria monitoring, and red blood cell antibodies will be drawn at baseline and at nominated times after malaria challenge.

Number of patients (planned): approximately20 volunteers (6 in cohort 1, 4 in cohort 2 and approximately 10 in cohort 3).

Diagnosis and main criteria for inclusion:

Inclusion Criteria

1) Volunteers will be males, aged between 18 and 45 years who do not live alone (between Day 1-5 or until admitted to the clinical unit).
2) Volunteers must have a BMI within the range 18–30.
3) Volunteers must understand the procedures involved and agree to participate in the study by giving fully informed, written consent.
4) Be contactable and available for the duration of the trial (maximum of 4weeks)
5) Volunteers must be non-smokers and in good health, as assessed during pre-study medical examination and by review of screening results.
6) Good peripheral venous access

Exclusion Criteria

1) History of malaria
2) Travelled to or lived (>2 weeks) in a malaria-endemic country during the past 12 months or planned travel to a malaria-endemic country during the course of the study
3) Has evidence of increased cardiovascular disease risk (defined as >10%, 5 year risk) as determined by the method of Gaziano et al., (15). Risk factors include sex, age, systolic blood pressure (mm Hg), smoking status, body mass index (BMI, kg/mm^2), reported diabetes status and blood pressure
4) History of splenectomy
5) History of a severe allergic reaction, anaphylaxis or convulsions following any vaccination or infusion.
6) Presence of current or suspected serious chronic diseases such as cardiac or autoimmune disease (HIV or other immunodeficiencies), insulin dependent diabetes, progressive neurological disease, severe malnutrition, acute or progressive hepatic disease, acute or progressive renal disease, psoriasis, rheumatoid arthritis, asthma, epilepsy or obsessive compulsive disorder, skin carcinoma excluding non spreadable skin cancers such as basal cell and squamous cell carcinoma
7) Known inherited genetic anomaly (known as cytogenetic disorders) e.g., Down’s syndrome
8) Volunteers wishing to be able to donate blood to the ARCBS in the future

9) Presence of retinal or visual field changes either attributable to 4-aminoquinoline compounds or to any other etiology
10) The volunteer has a diagnosis of schizophrenia, bi-polar disease, or other severe (disabling) chronic psychiatric diagnosis
11) The volunteer has been hospitalized within the past 5 years prior to enrollment for psychiatric illness, history of suicide attempt or confinement for danger to self or others
12) The volunteer is receiving psychiatric drugs. Participants who are receiving a single antidepressant drug and are stable for at least 3 months prior to enrollment without decompensating may be allowed to enroll in the study at the investigator's discretion.
13) Known pre-existing prolongation of the QTc interval
14) Family history of congenital prolongation of the QTc interval on electrocardiograms or of sudden death or any other clinical condition known to prolong the QTc interval, e.g. volunteers with a history of symptomatic cardiac arrhythmias, with clinically relevant bradycardia or with severe cardiac disease
15) Recent or current therapy with an antibiotic or drug with potential antimalarial activity (tetracycline, azthromycin, clindamycin, hydroxychloroquine etc.)
16) Known hypersensitivity to artemether or lumefantrine, atovaquone or proguanil hydrochloride (Malarone®), or any of the excipients (Microcrystalline cellulose (E460), croscarmellose sodium, magnesium stearate, hypromellose, anhydrous colloidal silica and polysorbate 80, hydroxypropylcellulose, povidone K30, sodium starch glycollate, macrogol 400, macrogol 8000, poloxamer 188 and pink colour concentrate OY-S-24972
17) Concomitant use of any drug which is metabolised by the cytochrome enzyme CYP2D6 (e.g. flecainide, metoprolol, imipramine, amitriptyline, clomipramine) OR drugs that are known to prolong the QTc interval, e.g. antiarrhythmics of classes IA and III, neuroleptics, antidepressant agents, certain antibiotics (including some agents of the following classes: macrolides, fluoroquinolones, imidazole and triazole antifungal agents), certain nonsedating antihistamines (terfenadine, astemizole), cisapride.
18) Use of corticosteroids, anti-inflammatory drugs, any immunomodulators or anticoagulants. Currently receiving or have previously received immunosuppressive therapy, including systemic steroids including ACTH or inhaled steroids in dosages which are associated with hypothalamic-pituitary-adrenal axis suppression such as 1mg/kg/day of prednisone or its equivalent or chronic use of inhaled high potency corticosteroids (budesonide 800 µg per day or fluticasone 750 µg)
19) Presence of acute infectious disease or fever (e.g., sub-lingual temperature ≥ 38.5°C) within the five days prior to study product administration)
20) Evidence of acute illness within the four weeks before trial prior to screening
21) Significant intercurrent disease of any type, in particular liver, renal, cardiac, pulmonary, neurologic, rheumatologic, or autoimmune disease by history, physical examination, and/or laboratory studies including urinalysis
22) Alcohol consumption greater than community norms (i.e. more than 21 standard drinks per week for males)
23) A history of drug habituation, or any prior intravenous usage of an illicit substance
24) Medical requirement for intravenous immunoglobulin or blood transfusions
25) Participation in any investigational product study within the 8 weeks preceding the study
26) Participation in any research study involving significant blood sampling, or blood donation to Red Cross (or other) blood bank during the 8 weeks preceding the reference drug dose in the study
27) Have ever received a blood transfusion
28) Positive test for HIV, Hepatitis B, hepatitis C, Human T-cell Lymphotropic Virus I & II (HTLVI &

HTLVII), and syphilis<br>29) Any clinically significant biochemical or haematologic abnormality (Hb must be ≥13.5g/dL)<br>30) Ingestion of any poppy seeds within the 48 hours prior to the screening blood test (volunteers will be advised by phone not to consume any poppy seed in this time period)<br>31) Detection of any drug listed Appendix 2 in the urine drug screen unless there is an explanation acceptable to the medical investigator (e.g. the subject has stated in advance that they consumed a prescription or OTC product which contained the detected drug) and/or the subject has a negative urine drug screen on retest by the pathology laboratory<br>32) Evidence of any condition that, in the opinion of the clinical investigator, might interfere with the evaluation of the study objectives or pose excessive risks to participants.
**Investigational product, dosage and mode of administration:**<br>**Reference treatments:**<br>**Riamet® tablets:** Artemether (20mg) and Lumefantrine (120mg): 4 tablets orally as a single dose twice a day with fatty food at time 0, 12, 24, 36, 48 and 60 hours, making a total dose of 24 tablets in 6 doses<br>**Malarone® tablets:** Atovaquone (250mg) and Proguanil hydrochloride (100mg), 4 tablets as a single dose daily for 3 consecutive days
**Criteria for evaluation:**<br>Safety: Clinical adverse events; hematology, chemistry, serology laboratory data; physical examination including vital signs and electrocardiograms (ECG).<br>Malaria specific: malaria PCR, blood thick films, clinical symptoms of malaria
**Statistical methods:**<br>This is a pilot study designed to assess the study design and estimate the variability of potential endpoints. A sample size of approximately 20 evaluable participants, with 10 participants in each therapy group, randomized 1: 1 of each therapy, is judged sufficient to estimate the descriptive statistics of the proposed endpoints (kinetics of clearance of parasitemia and parasite DNA).

LIST OF ABBREVIATIONS

ADCI	Antibody-Dependant Cellular Inhibition
AE	Adverse Event/Adverse Experience
AST	Aspartate aminotransferase
ALT	Alanine aminotransferase
BSPC	Blood Stage *Plasmodium falciparum* challenge inoculum
CMI	Cell Mediated Immunity
CRF	Case Report Form
CTMF	Clinical Trial Master File
FBC	Full Blood Count
GCP	Good Clinical Practice
GMP	Good Manufacturing Practice
HBV	Hepatitis B virus
HCV	Hepatitis C virus
HIV	Human immunodeficiency virus
HREC	Human Research Ethics Committee
IRB	Institutional Review Board
ICH	International Conference on Harmonisation
IM	Intramuscular
IP	Investigational Product
ISF	Investigator Site File
PI	Principal Investigator
QIMR	Queensland Institute of Medical Research
PBS	Phosphate-buffered saline
PCR	Polymerase Chain Reaction
SAE	Serious Adverse Event/Serious Adverse Experience
SRT	Safety Review Team
SOP	Standard Operating Procedure
WHO	World Health Organization

TABLE OF CONTENTS

1 BACKGROUND 1
1.1 Rationale for the study 1
1.2 Drug Development Strategy 1
1.3 Objective 2
2 STUDY DESIGN 2
3 INVESTIGATIONAL PRODUCT 2
3.1 Malaria Inoculum 2
3.2 Reference/Rescue Drug 3
3.3 Preparation 3
3.4 Packaging, labelling and storage 4
3.5 Product accountability 4
4 SUBJECT RECRUITMENT 5
4.1 Number of volunteers 5
4.2 Pre-study screening 5
4.3 Inclusion criteria 7
4.4 Exclusion criteria 7
5 STUDY PLAN and Procedures 10
5.1.1 Enrolment/Baseline 10
5.1.2 Medical and Compliance review/Randomisation 13
5.1.3 Dosing 13
5.1.4 Blood sampling 14
5.1.5 Safety measures 14
5.1.6 Meals and Fluid Restrictions 15
5.1.7 Concomitant Medications 15
5.1.8 Laboratory Safety Assessments 15
5.2 Withdrawal from treatment 15
5.3 Emergency procedures 16
5.4 Safety Oversight 16
6 ADVERSE EVENTS 16
6.1 Definitions 16
6.2 Causality 17
6.3 Adverse Event Severity – Definition 18
6.4 Treatment and Follow-up of Adverse Events 18
7 PREGNANCY 18
8 STATISTICS AND DATA MANAGEMENT 19
Objectives 19
8.1 Primary Objective 19
8.2 Secondary Objective 19
8.3 General Design 19
8.4 Data management 21

8.5 Description of Statistical methods to be employed 21
8.6 Analyses of Primary Objective 21
8.7 Analyses for Safety 21
8.7.1 Demographic and safety data 22

9 ETHICAL CONSIDERATIONS 22

9.1 Ethical principles 22
9.2 Ethical review 22
9.3 Subject information and consent 23
9.4 Subject data protection 23
9.5 Subject compensation 23

10 ADMINISTRATIVE DETAILS 23

10.1 Liability/indemnity/insurance 23
10.2 Changes to final study protocol 24
10.3 Clinical Data Recording 24
10.4 Record Retention 25
10.5 Biological Samples 25
10.6 Monitoring 25
10.7 Reporting and communication of results 25
10.8 Discontinuation of the study 25
10.9 Study audit 26

11 REFERENCES 26

12 SIGNED AGREEMENT TO STUDY PROTOCOL 27

APPENDICES

Appendix 1 Schedule of Events
Appendix 2 Laboratory Evaluations
Appendix 3 Medical History and Physical Examination
Appendix 4 Participant Information Leaflet and Informed Consent
Appendix 5 Symptoms and Signs of Malaria
Appendix 6 Preparation of Malaria Inoculum
Appendix 7 Riamet® and Malarone® Product Information & Consumer Information
Appendix 8 Assessment of Adverse Event Severity
Appendix 9 Severity Tables for Grading Laboratory Adverse Events
Appendix 10 Tables for Grading Adverse Events

1 BACKGROUND

1.1 Rationale for the study

The World Health Organization reported in 2005 that malaria kills more than 1 million people annually and that approximately 3.2 billion people living in 107 countries or territories are at risk of infection (1). Most of the malaria mortality occurs in sub-Saharan Africa and in children under 5 years of age. Of the four species of malaria parasite that infect humans, *Plasmodium falciparum* is responsible for the majority of these deaths. Mounting drug resistance of the malaria parasite, as well as widespread resistance of mosquitoes to insecticides, makes the current control strategies increasingly unrealistic. A drug that would reduce both mortality and morbidity secondary to *P. falciparum* infection would be a valuable resource in the fight against this disease.

P. falciparum has a complex life cycle. Sporozoites, the infectious stage of the parasite, are transmitted to humans through the saliva of infected female mosquitoes while taking a blood meal. Sporozoites travel through the bloodstream to the liver, where they invade hepatocytes and then multiply asexually into merozoites. Six to 10 days after invasion, the hepatocytes rupture, releasing thousands of merozoites into the bloodstream. These merozoites invade erythrocytes, multiply and after 2 days, release progeny merozoites, which subsequently invade new erythrocytes to continue the asexual blood-stage cycle. Clinical symptoms in humans are due to this asexual blood-stage of the parasite’s life cycle. A small percentage of merozoites do not multiply after invading erythrocytes, but instead differentiate into gametocytes. These gametocytes are ingested by a female mosquito during a subsequent blood meal and undergo sexual reproduction in the mosquito midgut, producing a zygote. The zygote matures and releases sporozoites that migrate to the mosquito’s salivary glands, thus completing the life cycle.

While the complex life-cycle of the malaria parasite offers several points that could be targeted by a drug, it is the lifecycle that occurs in the blood of infected participants that is responsible for all the pathology seen in malaria. Thus, new drugs aimed at killing blood stage parasites would be desirable.

1.2 Drug Development Strategy

Malaria drug development requires reliable means of selecting the best candidates for progression. While clinical efficacy trials in malaria-endemic areas represent the gold standard for establishing the protective efficacy of any antimalarial drug, the logistic challenges and expense of undertaking such trials in malaria-endemic areas will prohibit the systematic testing of the large number of therapeutic drugs under development. The approach to date to select promising drugs meriting progression to efficacy trials have generally included animal treatment studies, including primates with adapted *P. falciparum* strains.

The novelty of this trial design rests in the use of a blood stage challenge as a test of drug efficacy. The reduced level of parasitaemia in the infected participants could correlate directly to the therapeutic potential of different drugs for use in treatment and prevention of clinical manifestation of malaria infection. Moreover, this approach has been previously used with success by us and others (3, 4, 6) in vaccine studies.

1.3 Objective

Primary:

- To validate a human malaria challenge study design and assess appropriate endpoints for assessment of efficacy of new antimalarial drug candidates for the treatment of *Plasmodium falciparum.*

Secondary:

- To confirm the parasite growth curves after I.V. inoculation of healthy volunteers with *P. falciparum* blood stage parasites
- To establish the parasite clearance profiles by PCR after administration of fast and slow acting antimalarial drugs at a target parasitemia of ≥1,000 parasites/ml after inoculation with an experimental malaria challenge
- To assess the safety of an experimental malaria challenge

2 STUDY DESIGN

This is a single-center, controlled, randomized, study using a BSP inoculum challenge as a model to assess the activity of antimalarial agents. The study will be conducted in at least 2 cohorts (n = 6, n = 4 and possibly cohort 3 = n = 10). Cohort 2 and 3 will not commence until at least after day 12 of the previous cohort and review by the Safety Review Team. The volunteers will be randomized 1:1 to the two registered antimalarials. This is an enabling study using registered antimalarial drugs as reference treatments (one slow acting and one fast acting), aimed to inform trial design, endpoints and testing regimens for assessing new candidate antimalarial drugs in development.

3 INVESTIGATIONAL PRODUCT

3.1 Malaria Inoculum

The inoculum containing *Plasmodium falciparum* strain 3D7, has been derived from blood donated from a donor with clinical manifestation of malaria. The preparation of this challenge inoculum has been described in detail (3). Briefly, the cells were purified from a donor previously infected with *P. falciparum,* strain 3D7, via mosquito bites. Before the infection, the donor was extensively screened and no serologic evidences were found for the screened infectious agents with exception of seropositivity for Epstein-Barr virus and cytomegalovirus. However, the stored blood sample is PCR negative for both viruses, indicating absence of viral DNA.

Once the donor was microscopically positive for presence of malaria parasites, one unit of blood (500ml) was collected from the donor and processed to remove leucocytes. The processed blood was then mixed with glycerolyte 57 solution (Baxter, Deerfield, IL) and cryopreserved in ~1mL aliquots as previously described (3).

The inoculum used for BSP challenge in this study, will contain an estimated 1,800 infected erythrocytes diluted into 2 mL of Normal Saline for Injection

The infective inoculum will have been prepared from the cryopreserved infected blood aliquots prepared as previously described (3). Inocula for each challenge group will be

prepared from a single blood aliquot. Each dose of 2 mL will contain ~1,800 infected erythrocytes. The inoculum will be prepared aseptically, as outlined in appendix 6. The actual number of parasites inoculated will take into account the loss of viability resulting from cryopreservation, storage and thawing. Previous experience indicates that parasite viability following this process is ~30%, thus requiring ~5,300 infected erythrocytes per inoculum. The parasitemia in frozen vials of the cell bank is 212p/µL. Thus, each inoculation will contain 250 µL of the thawed sample which will contain about 12.5×10^8 erythrocytes and 5,300 infected erythrocytes . Following inoculation of each group of volunteers, an identical inoculum will be placed into tissue culture and subject to limiting dilution analysis, to accurately quantify the number of viable parasites inoculated in each group.

3.2 Reference/Rescue Drug

Preparation:

Riamet® (*20mg Artemether and 120mg Lumefantrine*) as tablets for oral use will be acquired by Q-Pharm.

Malarone ® *Atovaquone (250mg) and Proguanil hydrochloride (100mg),*

Administration:

Both drugs will be administrated orally in a tablet form as described in product insert.

Dosage:

Riamet: A course of treatment comprises six doses of four tablets (total course of 24 tablets) given over a period of 60 hours following food. The first dose, given at the time of initial diagnosis (or scheduled administration), should be followed by five further doses given at 12, 24, 36, 48, 60 hours.

Malarone: Given as 4 tablets in one dose with food, daily for 3 days.

If allergy or contraindication to Riamet or Malarone develops, chloroquine will be administered as the challenge strain, 3D7 is chloroquine-sensitive.

3.3 Preparation

Malaria Inoculum Preparation:

The inoculum will be prepared as per Appendix 6. Based on prior experience and parasite data, Cohort 1 received approximately 50 viable parasites. Briefly, the infected erythrocytes will be thawed and washed, resuspended in normal saline, diluted and dispensed into syringes. The inoculum will be kept on ice until injected. For Cohorts 2 and beyond, based on the cell count and the previously determined parasitaemia, a 250µL volume of the thawed sample which has been estimated to contain around 5,300 infected erythrocytes will be mixed with clinical grade saline. The total volume of the inoculum for injection will be 2 mL.

Administration

The inoculum containing around 5,300 *P. falciparum* infected erythrocytes will be administrated intravenously in all volunteers. All volunteers in each cohort will be inoculated intravenously within thirty (30) minutes period.

Volunteers will undergo intravenous cannulation with appropriate gauge butterfly cannula.

Placement and patency will be checked by flushing the vein with 5 mL of clinical grade saline. The inoculum will be injected intravenously, and the cannula again flushed with 5 mL of clinical grade saline. The cannula will then be removed, and haemostasis ensured by use of a crepe bandage.

Dosage: Each volunteer will receive a single dose of infectious inoculum on enrollment.

Reference/Trial Drug Preparation:
The trial/placebo and reference/rescue medications will be dispensed and accounted for in accordance with Q-Pharm standard procedures. All used medications will be fully documented.

3.4 Packaging, labelling and storage

Malaria Inoculum: On Day 0, the frozen blood aliquots will be thawed and used to prepare the challenge inocula. The time between thawing and inoculation will be maintained at maximum of 4 hours, during which time all inocula will be stored on ice. All volunteers will be challenged intravenously within 30 minutes period.

Reference Drug supplies will be provided to Q-Pharm, labeled according to identity, brand or source, and batch number. The supplies will be held in appropriate locked storage conditions at Q-Pharm until required. The contents of the label for drug to be administered to the volunteers will be in accordance with all applicable regulatory requirements.

3.5 Product accountability

The vials containing the inoculums of the blood stage parasites will be stored at QIMR or Q-Gen prior to initiation of the study. The Q-Pharm pharmacist will document receipt conditions and time restrictions of use.

The clinical site will acquire drug supplied from the sponsor or local pharmacy prior to the initiation of the study, when the approval has been obtained from relevant ethics committee.

Vials containing the drug control will be inventoried prior to the beginning of study enrollment on study accountability logs in regards to condition upon receipt, vial quantities, formulation/type of study product, lot and vial numbers. The investigator or qualified study person designated by the investigator will ensure that the received study products are the specified formulation. Each dose of study drug, or control used or wasted will be accounted for in writing on the study accountability log at the time of the removal of the product from storage. The site pharmacist or a nominee designated by the investigator is responsible for maintaining an accurate inventory and accountability record of drug/saline control supplies for this study. Partially used vials may not be administered to other volunteers.

Study products and study accountability logs will be available to the sponsor or sponsor's representative as part of the study monitoring procedures.

The reference medications will be dispensed and accounted for in accordance with Q-Pharm standard procedures. All used medications will be fully documented. Unused investigational product will be discarded following the conclusion of the study. The unused product will be subject to a final reconciliation of stock by the sponsor's monitor.

4 SUBJECT RECRUITMENT

For this study, healthy, non smoking male adult volunteers between 18-45 years of age will be enrolled. No restrictions will apply for ethnic or racial categories. The expected population to be enrolled from the database of healthy volunteers maintained by Q-Pharm and it may include all Australian racial categories, such as Australian White, Australian Indian, Australian Asian, Australian Aborigines or Torres Strait Islanders.

For the study, at least 10 to 20 volunteers will be recruited, and after providing written screening informed consent, will undergo eligibility screening, including medical history, physical examination including an ECG, laboratory investigations including haematology testing, liver and renal function tests, HIV, Hepatitis B and C screening and urinalysis, blood grouping and red cell antibody testing, CMV, EBV, Human T-cell Lymphotropic Virus I & II (HTLVI & HTLVII), and syphilis testing. It is estimated that up to 25-40 volunteers will need to be screened to complete enrolment.

Volunteers will be recruited from the HREC approved database of healthy volunteers maintained by Q-Pharm, or by a general or study specific advertisement via print, radio or poster media to students of Queensland universities or to the general community, as approved by the Queensland Institute of Medical Research Human Research Ethics Committee (QIMR-HREC).

4.1 Number of volunteers

This study is aimed to define the conditions for the malaria challenge design with known antimalarial agents, Riamet® or Malarone®.

Volunteers will initially be screened for eligibility for the study. Volunteers who attend the clinic for a recruiting medical interview will be allocated a screening number.

It is planned that of the at least 10 eligible volunteers, Cohort 1 of 6 volunteers and 2 reserves, and Cohort 2 of 4 volunteers and 2 reserves and possibly cohort 3 of 10 volunteers and 2 reserves, will attend the clinic on the study day 0 of each treatment period. If any of the nominated volunteers has ceased to be eligible (e.g. as a result of a protocol violation) or fails to appear or is unable to proceed, one or both of the reserve volunteers will be enrolled, to endeavor to enroll 6, 4 or possibly10 volunteers in each cohort to complete the dosing. The cohort 2 and 3 volunteers will attend the clinic at least after an INTERIM safety review after the day 9 results of the previous cohort. Volunteers who are dosed, but who fail to complete the study for any reason, may be replaced as agreed by the PI and the sponsor.

4.2 Pre-study screening

A Schedule of Events, which details all the procedures to be conducted during recruitment, (as well as during the confinement and post confinement periods) is located in Appendix 1.

A screening visit will be scheduled after an initial contact screen by clinical trial staff consisting of background information of the trial. During this initial screening visit, the subject will read the Participant Information Leaflet (Appendix 4) and be encouraged to ask questions.

Volunteers willing to be considered for inclusion may sign the screening consent form during the screening visit, or return after further consideration. The volunteer will be given a copy of the consent for their records. The signed and dated originals will be held on file by Q-Pharm.

After providing written consent to participate, the volunteer will be examined by the medical investigator and physical examinations, vital sign and ECG testing will be done together with safety blood and urine assessments will be obtained. The volunteers will be fully informed of the nature of the study at this time, and the requirement to repeat some screening tests if indicated (vital signs, urine drug screening) on the day of confinement to determine their continuing eligibility.

The pre-study screening will be conducted within four weeks prior to the first scheduled dosing day and will include.

<u>Procedures to be followed for screening:</u>

1. Explain the study via the Participation Information Leaflet (Appendix 4) and gain Informed Consent from the volunteer.
2. Ensure the subject has signed the Participation Information Leaflet and consent and received a signed copy.
3. Elicit a complete medical history.
4. Elicit a social history including alcohol use.
5. Undertake a complete physical examination.
6. Assessment of the 5 year cardiovascular event risk based on the method published by Gaziano et al, (15). The risk factors assessed will include sex, age, body mass index, blood pressure, history of diabetes mellitus and history of smoking.
7. Obtain a 12 lead ECG
8. Obtain minimum of 20 mL of blood for hematology, biochemistry and serologic tests for viral hepatitis B and C, HIV, HTLV 1&2, CMV, EBV and syphilis in all subjects. (Appendix 2)
9. Urine collection for urinalysis and urine drug screen.
10. Verify subject meets inclusion/exclusion criteria.
11. A screening number will be assigned to each subject.

Volunteers who complete all screening procedures and satisfy all entry criteria will be considered eligible to participate in this study. To be eligible for study entry, clinical laboratory values at screening must not be clinically significantly outside the range of the normal values. Re-screening will not be allowed unless the Medical Investigator considers the cause of the initial pre-screening failure to be of an acute and completely reversible nature.

If screening laboratory results are abnormal, e.g., HIV testing, the volunteer will be referred for appropriate counseling. If any clinically significant abnormalities are detected during screening, the participant will be referred for follow-up tests to a general practitioner or medical specialist as appropriate.

4.3 Inclusion criteria

1) Volunteers will be males, aged between 18 and 45 years who do not live alone (from Day 1 until at least the end of the antimalarial drug treatment).
2) Volunteers must have a BMI within the range 18–30.
3) Volunteers must understand the procedures involved and agree to participate in the study by giving fully informed, written consent.
4) Be contactable and available for the duration of the trial (maximum of 4weeks)
5) Volunteers must be non-smokers and in good health, as assessed during pre-study medical examination and by review of screening results.
6) Good peripheral venous access

4.4 Exclusion criteria

Participants may be excluded from the study either during screening, on blood stage challenge days, or during the blood sampling intervals, for any of the following reasons:

1) History of malaria
2) Travelled to or lived (>2 weeks) in a malaria-endemic country during the past 12 months or planned travel to a malaria-endemic country during the course of the study
3) Has evidence of increased cardiovascular disease risk (defined as >10%, 5 year risk) as determined by the method of Gaziano et al., (15). Risk factors include sex, age, systolic blood pressure (mm Hg), smoking status, body mass index (BMI, kg/mm^2), reported diabetes status and blood pressure.
4) History of splenectomy
5) History of a severe allergic reaction, anaphylaxis or convulsions following any vaccination or infusion.
6) Presence of current or suspected serious chronic diseases such as cardiac or autoimmune disease (HIV or other immunodeficiencies), insulin dependent diabetes, progressive neurological disease, severe malnutrition, acute or progressive hepatic disease, acute or progressive renal disease, psoriasis, rheumatoid arthritis, asthma, epilepsy or obsessive compulsive disorder, skin carcinoma excluding non spreadable skin cancers such as basal cell and squamous cell carcinoma
7) Known inherited genetic anomaly (known as cytogenetic disorders) e.g., Down's syndrome
8) Volunteers wishing to be able to donate blood to the ARCBS in the future
9) Presence of retinal or visual field changes either attributable to 4-aminoquinoline compounds or to any other etiology
10) The volunteer has a diagnosis of schizophrenia, bi-polar disease, or other severe (disabling) chronic psychiatric diagnosis
11) The volunteer has been hospitalized within the past 5 years prior to enrollment for psychiatric illness, history of suicide attempt or confinement for danger to self or others

12) The volunteer is receiving psychiatric[1] drugs. Participants who are receiving a single antidepressant drug and are stable for at least 3 months prior to enrollment without decompensating may be allowed to enroll in the study at the investigator's discretion.
13) Known pre-existing prolongation of the QTc interval
14) Family history of congenital prolongation of the QTc interval on electrocardiograms or of sudden death or any other clinical condition known to prolong the QTc interval, e.g. volunteers with a history of symptomatic cardiac arrhythmias, with clinically relevant bradycardia or with severe cardiac disease
15) Recent or current therapy with an antibiotic or drug with potential antimalarial activity (tetracycline, azthromycin, clindamycin, hydroxychloroquine etc.)
16) Known hypersensitivity to artemether, lumefantrine, atovaquone or proguanil hydrochloride, or any of the excipients (Microcrystalline cellulose (E460), croscarmellose sodium, magnesium stearate, hypromellose, anhydrous colloidal silica and polysorbate 80, hydroxypropylcellulose, povidone K30, sodium starch glycollate, macrogol 400, macrogol 8000, poloxamer 188 and pink colour concentrate OY-S-24972
17) Concomitant use of any drug which is metabolised by the cytochrome enzyme CYP2D6 (e.g. flecainide, metoprolol, imipramine, amitriptyline, clomipramine) OR drugs that are known to prolong the QTc interval, e.g. antiarrhythmics of classes IA and III, neuroleptics, antidepressant agents, certain antibiotics (including some agents of the following classes: macrolides, fluoroquinolones, imidazole and triazole antifungal agents), certain nonsedating antihistamines (terfenadine, astemizole), cisapride.
18) Use of corticosteroids, anti-inflammatory drugs, any immunomodulators or anticoagulants. Currently receiving or have previously received immunosuppressive therapy, including systemic steroids including ACTH or inhaled steroids in dosages which are associated with hypothalamic-pituitary-adrenal axis suppression such as 1mg/kg/day of prednisone or its equivalent or chronic use of inhaled high potency corticosteroids (budesonide 800 µg per day or fluticasone 750 µg)
19) Presence of acute infectious disease or fever (e.g., sub-lingual temperature ≥ 38.5°C) within the five days prior to study product administration
20) Evidence of acute illness within the four weeks before trial prior to screening
21) Significant intercurrent disease of any type, in particular liver, renal, cardiac, pulmonary, neurologic, rheumatologic, or autoimmune disease by history, physical examination, and/or laboratory studies including urinalysis
22) Alcohol consumption greater than community norms (i.e. more than 21 standard drinks per week for males)
23) A history of drug habituation, or any prior intravenous usage of an illicit substance
24) Medical requirement for intravenous immunoglobulin or blood transfusions
25) Participation in any investigational product study within the 8 weeks preceding the study
26) Participation in any research study involving significant blood sampling, or blood donation to Red Cross (or other) blood bank during the 8 weeks preceding the reference drug dose in the study

27) Have ever received a blood transfusion
28) Positive test for HIV, Hepatitis B, hepatitis C, Human T-cell Lymphotropic Virus I & II (HTLVI & HTLVII), and syphilis
29) Any clinically significant biochemical or haematologic abnormality (Hb must be ≥13.5g/dL)
30) Ingestion of any poppy seeds within the 48 hours prior to the screening blood test (volunteers will be advised by phone not to consume any poppy seeds in this time period)
31) Detection of any drug listed Appendix 2 in the urine drug screen unless there is an explanation acceptable to the medical investigator (e.g. the subject has stated in advance that they consumed a prescription or OTC product which contained the detected drug) and/or the subject has a negative urine drug screen on retest by the pathology laboratory
32) Evidence of any condition that, in the opinion of the clinical investigator, might interfere with the evaluation of the study objectives or pose excessive risks to participants.

[1]aripiprazole, clozapine, ziprasidone, haloperidol, molindone, loxapine, thioridazine, thiothixene, pimozide, fluphenazine, risperidone, mesoridazine, quetiapine, trifluoperazine, trifluopromazine, chlorprothixene, chlorpromazine, perphenazine, olanzapine, carbamazepine, divalproex sodium, lithium carbonate or lithium citrate.

(b) On dosing days, and during the blood collection intervals:

1) Ingestion of any Riamet or Malarone related drug since the recruitment interview (other than the doses administered in this study).
2) Ingestion of any other drug, including St John's Wort, in the week prior to dosing or during the blood sampling period which, in the opinion of the Medical Investigator, could compromise the study e.g. through pharmacokinetic or metabolic interactions, or analytical interference. However the Medical Investigator may permit the use of paracetamol for the treatment of headache or other pain. If drug therapy other than paracetamol or drug specified in the protocol, is required during the study periods, a decision to continue or discontinue the subject's participation will be made by the Medical Investigator, based on the nature of the medication and the time the medication was taken. (Note: Oral, injectable or implant contraceptive for female volunteers is acceptable).
3) Failure to conform to the requirements of the protocol.
4) Detection of any drug listed in Appendix 2 in the urine drug screen unless there is an explanation acceptable to the medical investigator (e.g. the subject has stated in advance that they consumed a prescription or OTC product which contained the detected drug).
5) Vital signs outside the reference range and clinically significant

Volunteers are requested to refrain from taking non-approved concomitant medication from recruitment until the conclusion of the study.

Volunteers who are excluded from participation on study days for any of the reasons may be eligible to participate on a postponed schedule if the Medical Investigator considers this appropriate.

5 STUDY PLAN AND PROCEDURES

A Schedule of Events, which details all the procedures to be conducted during recruitment, confinement and post confinement, is located in Appendix 1.

5.1.1 Enrolment/Baseline

Participation consent must be obtained from all eligible volunteers prior to enrollment. Volunteer must confirm that they will not be living alone on release from the day when they receive the inoculum until confinement at the clinical site and until finishing the treatment of antimalarial drugs. On the day of challenge (study day 0), extra volunteers may be invited to participate than are scheduled to be challenged. These alternates will be compensated for the study visit even if not inoculated, as described in the participation information and consent form.

Following blood stage challenge with *P. falciparum* in humans, the pre-patent period (interval between inoculation and appearance of parasites in the blood) as detected by PCR and blood smear has been reported to range from ≥ 3 days and ≥ 7 days respectively (6).

Study Day 0
(**CHALLENGE INOCULUM DOSE**)

1. Verify that all applicable eligibility criteria have been met.
2. Medical Investigator to perform medical history and physical examination, to assess eligibility to enter study.
3. Record vital signs
   (blood pressure, temperature, heart rate, and respiratory rate).
4. Obtain a 12 lead ECG
5. Urine collection for urine drug screen.
6. Perform breath alcohol test
7. Volunteers will be cannulated with an indwelling intravenous cannula for the malaria inoculum, and record which arm utilized.
8. Obtain approximately 30 mL of blood for baseline hematology (FBC and differential, malaria thick film assessment), biochemistry (including electrolytes creatinine, liver function tests), and blood for study of red cell alloantibody and safety serum storage
9. Administer the malaria inoculum

10. Observe for minimum of 60 minutes after administration of the inoculum to evaluate for immediate adverse reactions. Vital signs will be repeated at 60 minutes and prior to leaving the clinic, approximately 4 hours after dosing.
11. Education of volunteer by study staff during the 4 hour post-inoculum interval, on the description of signs or symptoms of malaria (Appendix 5). Emphasize to volunteer the importance of returning on Day 5 PM or as advised by the clinical staff for malaria treatment during confinement.
12. Record adverse events and concomitant medications.

Study Day 1 and 2 post challenge

During this period, volunteers are expected to be asymptomatic and aparasitemic, as determined by PCR

1. A phone call will be made to the volunteers during the day to solicit any adverse events.

Study Day 3-4 AM and PM and Day 5 AM

Follow-up from day three until the end of day 5 post challenge will be undertaken through twice daily visits to the clinical site for clinical evaluation and blood sampling. An experienced nurse will be in attendance at the study centre throughout this period and the Medical Investigator will be available within 30 minutes callback if required.

1. Volunteers will be reviewed each morning (approximately 8am) and evening (approximately 8pm) for blood sampling and clinical assessment.
2. Record vital signs (blood pressure, temperature, heart rate, and respiratory rate).
3. Medical Investigator to perform abbreviated physical examination (Appendix 3), to assess for any symptoms or signs of malaria (Appendix 5)
4. Obtain approximately 4 mL of blood for malaria PCR and thick film assessment for malaria.

Study Day 5 PM – Day 9 AM
(INPATIENT OBSERVATION AND TREATMENT PHASE)

To ensure volunteers safety, from the evening of day 5 post challenge or the first PCR parasitaemia of ≥1,000 parasites/mL, volunteers will be admitted overnight to the residential unit at the Q-Pharm clinical trials facility to facilitate close monitoring for clinical features of malaria, and twice daily blood clinical assessments and collection for malaria smears. If the PCR is negative the volunteers will be asked to return the following morning for further monitoring. Volunteers will be required to report to the study centre for confinement at approximately 07:00- 08:00 pm on the Day 5 evening or as advised by the clinical staff and as outlined below remain at the study centre for at least 3 consecutive days. An experienced nurse will be in attendance at the study centre throughout this period and the Medical Investigator will be available within 30 minutes callback.

If the volunteer develops a positive blood thick film, platelet count less than 100 x 10^9/L, or

malaria symptoms, or PCR parasite quantification of ≥1,000 parasites/mL before Day 5 PM, then the volunteer will be admitted in the clinic for observation and treatment as soon as the symptoms of malaria infection are confirmed and the volunteer will be treated with his allocated antimalaria treatment drug according to the randomization.

All positive blood films will be confirmed by at least one other microscopist with experience in the identification of malaria. Following treatment, daily blood films will continue until three consecutive smears are negative. Blood films will be saved for later re-examination. FBC and serum biochemistry will be performed, as clinically indicated when parasites are detected, and will be repeated as needed to confirm resolution of any significant laboratory abnormalities.

1. Volunteers will report to the unit at approximately 7-8pm on Day 5 post challenge or as advised by the clinical staff.
2. Medical Investigator to perform abbreviated physical examination, to assess any signs or symptoms of malaria
3. An ECG will be conducted on admission to the unit.
4. Record vital signs (blood pressure, temperature, heart rate, and respiratory rate).
5. Obtain approximately 25 mL of blood for malaria PCR and malaria thick film assessment and safety bloods.
6. Obtain urine samples for urine drug screen on admission to the unit.
7. Perform breath alcohol test
8. Volunteers will be monitored as above for symptoms or signs of malaria and have blood collected for malaria testing and monitoring of the platelet count each morning and evening for the duration of the confinement period.
9. When PCR parasite quantification is ≥1,000 parasite/mL OR a volunteer develops either symptoms or signs of malaria (Appendix 5), OR has a platelet count less than 100 x10^9/L, OR their blood shows ≥2 malaria parasites on thick smear (observed ~ day 7-9 in previous blood stage challenge studies (10)) the volunteer will receive the allocated antimalarial. The antimalarial treatments will be dosed according to randomization and administered as instructed by their manufacturers.

   All oral antimalarial drugs will be administered under direct observation. In the rare event that a subject requires hospitalization, this will be done at the Infectious Diseases Unit, Royal Brisbane and Women's Hospital.
10. Following the treatment, continue with the collection of blood samples (4mL) morning and evenings for three days (Day 7–9) for measurement of the parasites in the blood. From Day 6 PM until end of Day 7 or for 24 hours from when the treatment was initiated, collect 4mL blood samples every 6 hours to closely monitor the number of parasites in the blood.
11. Volunteers will be allowed to leave the unit either on Day 9 morning following medical examination, blood collection for safety assessment and malaria monitoring. OR at the discretion of the clinical investigator 36 hours after treatment has been initiated if they are clinically asymptomatic, with normal examination and tolerating antimalarial treatment.

It is anticipated that antimalarial treatment will be curative; recrudescence has not occurred in any of the previous challenge studies. In the unlikely event that malaria recurs, the individual will be retreated with an alternative regimen if appropriate.

Study Day 9 PM – Day 12 AM
(OUT PATIENT POST-TREATMENT PHASE)
From discharge until the day 12 or 4 days after completion of the antimalarial treatment, follow-up will be undertaken through twice daily visits (day 9 and 10) and once per day visit (day 11 and 12) to the clinical site for clinical evaluation and blood sampling.

1. Volunteers will be reviewed in the evening of day 9 (approximately 8pm), morning (approximately 8am) and evening (approximately 8pm) on Day 10, and morning ONLY for Day 11 and Day 12 for blood sampling and clinical assessment.
2. Record vital signs (blood pressure, temperature, heart rate, and respiratory rate).
3. Obtain approximately 4 mL of blood for malaria PCR and thick film assessment for malaria on each visit.

Study Day 28 OR End of Study (study withdrawal)
(FINAL VISIT)
1. Medical Investigator to perform medical history and physical examination
2. Record vital signs
   (blood pressure, temperature, heart rate, and respiratory rate).
3. Obtain a 12 lead ECG
4. Collect urine sample for urinalysis
5. Obtain approximately 30 mL of blood for hematology (FBC and differential, red-cell antibody), biochemistry (electrolytes creatinine, liver function tests), and blood for study of red cell alloantibody and safety serum storage and urine will be collected for assessment of urinalysis.

5.1.2 Medical and Compliance review/Randomisation

On admission to the study centre, volunteers will be required to undertake further screening procedures, including those laboratory tests specified in Appendix 2, to determine whether they remain eligible to be enrolled.

On the Day 0, malaria inoculation day, the Medical Investigator will review all volunteers' screening results prior to their randomization into the study. The Medical Investigator will emphasize the requirement to return for malaria drug treatment after the malaria inoculation. In the following period, volunteers will be reviewed by the Medical Investigator to confirm their continued eligibility for the study.

5.1.3 Dosing

The Blood Stage *Plasmodium falciparum* challenge inoculum will be prepared in Q-Gen on Day 0, according to Appendix 6 by QIMR nominated staff under guidance of the PI. All volunteers in each cohort will receive the malaria inoculum (BSPC) within 30 minutes period. Doses will be administered at approximately 8 am under the supervision of the Medical Investigator.

For treatment of malaria volunteers will be dosed according to the randomization schedule of either Riamet® or Malarone®. Volunteers will be instructed to swallow the 4 tablets whole, without biting or chewing, and to follow this with a full cup of water (200 mL). The Riamet® is given following food at time 0 h, 12, 24, 36, 48 and 60 h. Malarone® is given as 4 tablets in one dose with food, daily over three days. The clinic staff will confirm compliance with the dosing instructions by conducting a visual inspection of the hands and oral cavity after dosing the subjects. The time of dosing will be recorded.

5.1.4 Blood sampling

Cannulation
Volunteers will be cannulated with an intravenous cannula and a pre-dose blood sample will be collected according to Appendix 1.

Q-Pharm's standard work instructions will apply to the allowed time windows.

The blood will be collected into tubes containing the appropriate anticoagulant. Samples will be processed according to the laboratory requirements.

5.1.5 Safety measures

Vital signs (temperature, heart rate, blood pressure and respiratory rate) will be measured on entry to the unit on a minimum of a daily basis at Day 0, and from Day 3-Day 12, according to the Appendix 1 schedule of events.

If the observation time and blood sampling time coincide, for precision of timing, blood collection will take precedence over other procedures scheduled at the same time. With regard to time windows allowance for study procedures Q-Pharm's standard work instructions will apply.

Volunteers may be quietly ambulant within the unit.

The Medical Investigator and/or an experienced nurse will be in attendance at the study centre throughout this period and the Medical Investigator will be available within 30 minutes callback if required. The PI or infectious disease clinician, sub-investigator will monitor the volunteers during the confinement period in the morning and evening and in the outpatients visit at Day 0, Day 3-5 and the follow up Day 28 visit.

Volunteers will be under observation and adverse events (if any) will be recorded and dealt with appropriately.

At the post-confinement visits, volunteers will again be given the opportunity to mention any problems, and will be asked non-leading questions regarding their general well-being and medication intake.

5.1.6 Meals and Fluid Restrictions

On Day 0, malaria inoculum, volunteers may be given breakfast at least half an hour prior to dosing. On the admission of the volunteers into the Q-Pharm unit on Day 5PM or as advised by the clinical staff, volunteers will be given a meal. Standard meals will also be supplied whilst in the clinic unit during confinement. Volunteers may drink water as desired. The clinic staff will ensure that volunteers maintain their fluid intake throughout the period of confinement. Volunteers may drink non-alcoholic, non-xanthine containing beverages as desired.

5.1.7 Concomitant Medications

On admission, volunteers will be questioned in relation to relevant aspects of compliance with the study protocol, including drug intake since their screening clinic visit. Details of all other drugs taken (prescription and over-the-counter) will be recorded at this time and appropriate action taken.

Any medication taken during the study for treatment of a medical condition or adverse event is to be recorded in the concomitant medication pages in the CRF

5.1.8 Laboratory Safety Assessments

Urine drug screen. At screening and on admission (day 0 and day 5PM) , all volunteers will be required to provide a urine sample which will be subjected to a drug screen, at the Q-Pharm clinic, which can detect the drugs listed in Appendix 2. If the result of the test is positive volunteers may be allowed to continue, or may be delayed or withdrawn according to site specific instructions. This will also include an alcohol breath test on the admissions (day 0 and Day 5PM).

Blood test.: At the final visit (Day 28 or end of study), additional blood will be taken from all volunteers for laboratory safety tests (ELFT and haematology screen, serology and red cell alloantibody screen per Appendix 2). Any significant deviations from results obtained during screening will be followed until resolution or investigated fully.

Malaria Monitoring: Blood will be collected per schedule for malaria assessment by blood thick film (which includes an FBC) and PCR. Blood Thick film results ≥2 parasites per film indicate that the volunteer should be treated.

5.2 Withdrawal from treatment

Volunteers are free to withdraw from the study at any time. Volunteers may also be withdrawn by the investigators. Possible reasons for withdrawal by the investigators include the occurrence of a serious adverse event, or failure by the subject to comply with the requirements of the protocol. The reason for withdrawal should be clearly recorded in the subject's Q-Pharm Clinic File and CRF.

If a volunteer withdraws or is withdrawn by the investigator a final blood sample will be taken for biochemistry and haematology screens and other end of study safety assessments. ***In***

addition, volunteers are informed on the essential requirement to complete the antimalarial drug treatment for their safety, via the Patient Information leaflet

5.3 Emergency procedures

Emergency procedures are in place at the Q-Pharm clinics for dealing with any unforeseen clinical emergencies which may arise. The Medical Investigator and/or an experienced nurse will be present at all times when volunteers are at the Centre.

5.4 Safety Oversight

Safety Review Team (SRT)

The SRT will consist of the Principal Investigator (or delegate), the Medical Director of MMV (or delegate) and at least one independent qualified physician.

The SRT will provide on-going oversight of the study to ensure that volunteer safety is maintained. The team will review the safety data following each treatment group and their recommendations could include the following:

- Suggest changes to the protocol if required for participant safety
- Ask for further investigation into specific events
- Stop or postpone the study
- Approve continuation of the study to the subsequent group

There will be a written safety review plan which will specify the members of the safety review team (by name), the frequency of review and the specific data that will be reviewed at each meeting.

6 ADVERSE EVENTS

It is the responsibility of the Principal Investigator to ensure that adverse events which occurring the context of the study are reported and documented.

6.1 Definitions

An adverse event is any untoward medical occurrence in a patient or clinical investigational subject administered a pharmaceutical product and which does not necessarily have to have a causal relationship with the treatment.

An adverse event can therefore be any unfavourable and unintended sign (including an abnormal laboratory finding), symptom or disease temporally associated with the use of a medicinal product, whether or not considered related to the medicinal product.

Pre-existing conditions, which worsen during a study, are to be reported as adverse events. They can become Serious Adverse Events if they fulfill one of the seriousness criteria listed below.

In this study, adverse events will be recorded and reported from subject randomization to

study completion for all volunteers who receive the challenge inoculum dose on day 0.

Medical events are any changes that may occur in the subject's medical condition (illness, signs or symptoms) between their screening examination and randomization into the study.

Serious Adverse Events

Serious adverse events include:

- death
- life-threatening events
- events causing persistent or significant disability or incapacity
- events requiring or prolonging inpatient hospitalization
- a congenital anomaly/birth defect
- any other event considered clinically/medically significant

Serious adverse events will be reported by the Principal Investigator to the sponsor's monitor within 24 hours. The 24 hour emergency contact details are listed on page III of this protocol.

Serious adverse events will also be reported, as soon as possible but in any case within the required time frame, to:

- the Therapeutic Goods Administration (the responsibility of the sponsor)
- the Human Research Ethics Committee of the Queensland Institute of Medical Research (the responsibility of the Investigator).

6.2 Causality

The investigators will decide if adverse events are related to the administered drug or the malaria inoculum (Investigational Product). The assessment of causality will be made using the following definitions:

Unrelated
This category is applicable to those adverse events which are judged to be clearly and incontrovertibly due to extraneous causes (disease, environment, etc.) and do not meet the criteria for the relationship listed under *Unlikely, Possible* or *Probable*.

Unlikely
In general, this category is applicable to an adverse event which meets the following criteria (must have the first two):

1. It does **not** follow a reasonable temporal sequence from administration of the IP

2. It may readily have been produced by the subject's clinical state, environment or toxic factors, or other modes of therapy administered to the subject.

3. It does not follow a known pattern of response to the IP.

4. It does not reappear or worsen when the IP is re-administered.

Possible

This category applies to those adverse events in which the connection with the IP administration appears unlikely but cannot be ruled out with certainty. An adverse event may be considered possible if, or when (must have the first two):

1. It follows a reasonable temporal sequence from administration of the IP.
2. It may have been produced by the subject's clinical state, environment or toxic factors, or other modes of therapy administered to the subject.
3. It follows a known pattern of response to the IP.

Probable

This category applies to those adverse events, which are considered, with a high degree of certainty, to be related to the IP. An adverse event may be considered probable, if (must have the first three):

1. It follows a reasonable temporal sequence from administration of the IP.
2. It cannot be reasonably explained by the known characteristics of the subject's clinical state, environment or toxic factors, or other modes of therapy administered to the subject.
3. It disappears or decreases on cessation or reduction in dose.
4. It follows a known pattern of response to the IP.
5. It reappears on re-challenge.

6.3 Adverse Event Severity – Definition

The severity of adverse events will be graded on a three point scale:

Mild: discomfort noted, but no disruption to normal daily activities

Moderate: discomfort sufficient to reduce or affect normal daily activities

Severe: inability to work or perform normal daily activities.

6.4 Treatment and Follow-up of Adverse Events

All adverse events will be documented in the Subject's CRF, and will be categorized according to their causality and severity and whether they are defined as a serious or non-serious adverse event. All adverse events will be followed until they are either resolved or adequately explained.

7 PREGNANCY

Volunteers should be instructed to notify the investigator if it is determined after completion of the study that their partner became pregnant within 4 weeks of study drug administration.

Whenever possible, a pregnancy should be followed to term, any premature termination reported, and the status of the mother and child should be reported to MMV after delivery, and the progress of the child should be followed and reported to the MMV for at least 6 months after delivery.

8 STATISTICS AND DATA MANAGEMENT

8.1 Objectives

8.2 Primary Objective

- To validate a human malaria challenge study design and assess appropriate endpoints for assessment of efficacy of new antimalarial drug candidates for the treatment of *Plasmodium falciparum.*

8.3 Secondary Objective

- To confirm the parasite growth curves after I.V. inoculation of healthy volunteers with *P. falciparum* blood stage parasites
- To establish the parasite clearance profiles by PCR after administration of fast and slow acting antimalarial drugs at a target parasitemia of ≥1,000 parasites/ml after inoculation with an experimental malaria challenge

- To assess the safety of an experimental malaria challenge

8.4 General Design

Justification for sample size chosen for Blood Stage Parasite Challenge study

The first essential characteristic of the blood stage parasite challenge protocol is the requirement that all volunteers challenged with the inoculum develop parasitaemia following challenge. Any measure of recovery subsequent to treatment is conditional on the disease being present for each volunteer. The pilot study will confirm that all participants develop parasitaemia following challenge with around 5,300 infected erythrocytes, and that the protocol can be safely and successfully executed.

Four studies have been conducted using blood stage parasites challenge (5, 8, 9, 10). Collectively 28 participants have developed parasitaemia after challenge with parasite inocula up to 6000 infected erythrocytes using the same protocol as proposed here. No participant has failed to develop parasitaemia following challenge. Using these data, the maximum possible failure rate for a specified sample size has been calculated by recursive application of the negative binomial distribution until a required confidence level is achieved.

The following table displays a range of confidence values, from 50% to 99.9%, for achieving parasitemia in all volunteers. It first sets out the maximum failure rate for each of these confidence levels based on the existing data from 28 participants (column 2). The subsequent columns display the change in calculated maximum failure rates (%) that will occur with 4, 8 or 16 additional participants, assuming that all participants develop parasitaemia. For example

with an additional 16 extra participants, as proposed in this study, the maximum failure rate would be 7% at a confidence level of 95%.

Table: Maximum failure rate of malaria challenge (%) for typical sample sizes at a range of confidence levels when all respondents are positive.

Confidence that all volunteers develop parasitemia after challenge (%)	Maximum failure rate (%)			
	n=28 (current dataset)	Sample size in addition to established data		
		n=28+4	n=28+8	n=28+16
50	2	2	2	2
80	6	5	4	4
90	8	7	6	5
95	10	9	8	7
99	15	13	12	10
99.9	22	19	17	15

These data demonstrate that: i) the existing data provide a reasonably robust estimate of the reliability of this blood stage parasite challenge protocol; ii) the change in confidence and upper limit of probability of failure of blood stage challenge to result in parasitemia will not vary appreciably across the range of feasible sample sizes for the pilot study. It is therefore judged that other considerations, including, power to differentiate different parasite DNA clearance kinetics, and safety and logistic issues should be the primary considerations in selection of the number of volunteers to be included in the study.

Parasite DNA clearance kinetics

To evaluate candidate drugs for treatment of malaria, a group of healthy volunteers from the target population are first infected with low dose of parasite, then treated with the experimental drug and finally assessed by clinical, parasitologic and molecular methods for evidence of abrogation of malaria infection. Because of cost and human welfare issues it is highly desirable that the smallest number of volunteers is subject to live challenge with malaria parasites.

A dataset of parasite clearance kinetics has been provided to us by Dr Rob Hersmen, The Radboud University Nijmegen Medical Centre. A series of regressions was run by Dr Peter O'Rourke, trial statistician to estimate the rate of decline in parasitaemia for each person in the dataset. The most parsimonious modelling used $y = \log_{10}$ parasitaemia and x = days since start of treatment with parasitaemia above the limit of detection. Data from the 7 people had reasonably similar slopes (0.846) and residual standard deviation (0.135), translating into a 7 fold decrease in parasitaemia per day. These values were then used to estimate required sample sizes for assumed meaningful differences from this mean value of 20%, 25% and 50%. The required group sizes for a two sided test at the 5% level of significance are 12, 8 and 3 respectively for 80% power and 15, 10 and 4 for 90% power. Hence, for the proposed sample size of 10 in each arm of the study an 80% power will be present to identify a difference of 25% as significant.

To maximize volunteer safety and ensure logistic capacity, at least two cohorts of 6 and 4 are proposed, with a Safety Review Team meeting interposed between the cohorts. Following the safety review after cohort 2, depending on the safety data of the administered inoculum accumulated in the previous cohorts, the statistical plan will be reviewed and further cohorts may added. Any amendments on the statistical plan will need to be approved by the QIMR/HREC and the sponsor before any changes are applied in the study.

8.5 Data management

Clinical and laboratory data will be managed according to the standard procedures of Q-Pharm, supplemented if required by any specific requirements of the sponsor.

8.6 Description of Statistical methods to be employed

This section briefly describes the statistical methods to be used. A detailed Statistical Analysis plan will fully describe the methods and will be finalized prior to lock of the study database. Any deviations from the statistical analysis plan will be documented and justified in the statistical report.

Estimates will be presented with their 95% confidence intervals. Analysis will involve both descriptive methods, and hypothesis driven significance testing. In particular, the decline in parasitaemia following drug treatment, and the adverse events for each of the participants will be presented as individual graphs.

8.7 Analyses of Primary Objective

A primary objective of this Phase 2 drug trial is to assess whether the therapeutic efficacy of an anti-malarial drug can be assessed by using direct challenge with a low dose of blood-stage parasites (BSP) and measuring the level of parasitaemia clearance. Firstly all volunteers will be assessed for development of parasitaemia following challenge. Modeling of clearance of parasitemia by PCR measurement of malaria parasitemia of multiple-daily blood samples will use linear regression of the log of parasitaemia against time since treatment. The gradient from this model, which is the logarithm of the clearance rate, will be the primary outcome variable for efficiency analyses.

Statistical analysis to compare the two groups of drug treated volunteers will use analysis of variance of the gradients estimated from the modeling of parasitaemia clearance rates. The cohort effect will be included to adjust the drug comparison for any differences between cohorts. The effect size will be presented along with its 95% confidence interval.

Secondary analyses will consider parasite clearance as proportions of volunteers achieving specific clearance rates, such as 10-fold clearance in 24 hours and 100-fold clearance in 48 hours. Parasite growth curves from challenge to start of treatment will be modeled by fitting sine wave functions as outlined by Bejon et al. and Sanderson et al.

8.8 Analyses for Safety

The safety and tolerability of blood stage challenge in healthy malaria-naive volunteers the incidence of AE and SAEs will be tabulated by treatment group (with pooled drug treatment

volunteers representing one group), System Organ Class and Preferred Term. Separate assessments of systemic and local reactions will be performed. The overall number and percentage of volunteers with at least one AE (and SAE) will be tabulated after each drug administration and over the entire study period.

Any clinically important deviations from normal occur in routine laboratory test results and/or vital signs will be listed.

Should the need arise for terminating the study early, the investigative team will discuss with the SRT the reason for termination.

8.8.1 Demographic and safety data

Demographic data will be summarized by descriptive statistics and will include total number of observations (n), mean, standard deviation (SD) and range for continuous variables and number and % with characteristics for dichotomous variables.

Clinical laboratory data (hematology, blood chemistry, and urinalysis) which is outside of the normal range will be listed in tables. Isolated laboratory abnormalities will be reported as AEs if they are considered to be clinically significant by the Investigator. Vital signs which are outside of the normal range and clinically significant will also be listed in tables. All adverse events will be listed by volunteer and will include details of the treatment received prior to onset, onset time, duration, severity and relationship to the study drug.

9 ETHICAL CONSIDERATIONS

9.1 Ethical principles

The study will be conducted in accordance with the principles of the Declaration of Helsinki (Recommendations guiding Medical Doctors in Biomedical Research Involving Human Subjects), and with the NH&MRC National Statement on Ethical Conduct in Human Research (2007). The conduct of the study will be in accordance with the Notes for Guidance on Good Clinical Practice (GCP) (CPMP/ICH/135/95), as adopted by the Australian Therapeutic Goods Administration (2000).

The Medical Investigator will take care to minimise any discomfort experienced by volunteers during these studies. The only invasive procedures will be blood collection by cannulation/venipunctures and inoculation. The maximum amount of blood to be collected from an individual in the study is approximately 200 mL (i.e less than a standard blood-bank donation but taken over at least a 5-8 week interval).

9.2 Ethical review

The protocol, consent forms and subject information sheets will be reviewed by the Queensland Institute of Medical Research - Human Research Ethics Committee (QIMR-HREC).

No study activities will be initiated prior to the approval of that Committee. All amendments and addenda to the protocol will similarly be submitted to the QIMR-HREC for prior approval.

9.3 Subject information and consent

Volunteers will be fully informed of the nature of the study, the properties and side effects of the investigational products, and all relevant aspects of study procedures in the 'Participant Information Leaflet' (Appendix 4). Volunteers will receive a copy of the 'Participant Information Leaflet' (Appendix 4) and the Consumer Medicine Information for Riamet® and Malarone®(Appendix 7). The nature of the study, the drug and its side effects will also be discussed with the volunteers by the Medical Investigator during recruitment. The volunteers may ask questions of the Medical Investigator or the Clinic Staff at any time.

The 'Informed Consent' will be signed and dated by the volunteers in the presence of an investigator. Volunteers will also be given a copy of and their signed 'Informed Consent'.

9.4 Subject data protection

Volunteers will be informed (Appendix 4) that their data are held on file by Q-Pharm, that these data may be viewed by staff of Q-Pharm (including, where necessary, staff of Q-Pharm other than the named investigators), and that data may also be sighted by the sponsor's monitor on behalf of the sponsor and by external auditors on behalf of either the sponsor or regulatory agencies at the Q-Pharm site only. They will similarly be informed that a report of the study will be submitted to the sponsor company and may also be submitted to government agencies and perhaps for publication, but that they will only be identified in such reports by their study identification number, initials and perhaps their gender and age. The investigators undertake to hold all personal information in confidence.

9.5 Subject compensation

Volunteers who complete the study will be paid $1400 compensation for their participation. Volunteers who withdraw or are withdrawn from the study will be compensated on a fractional basis for their involvement unless they are withdrawn as a consequence of their misconduct. Reserve volunteers who do not participate in the study will be paid $150 compensation for the inconvenience associated with their attendance for screening and for their attendance on the dosing day of period 1, in case they are required to participate.

10 ADMINISTRATIVE DETAILS

10.1 Liability/indemnity/insurance

The study sponsor will ensure sufficient insurance is available to enable it to indemnify and hold the investigator(s) and relevant staff as well as any hospital, institution, ethics committee or the like, harmless from any claims for damages for unexpected injuries, including death, that may be caused by the subject's participation in the study but only to the extent that the claim is not caused by the fault or negligence of the volunteers or investigator(s). The sponsor

adheres to the guidelines of Medicines Australia for injury resulting from participation in a company sponsored trial, including the provision of "No-fault clinical trial insurance".

10.2 Changes to final study protocol

Changes to the final study protocol can only be made with the prior consent of the Principal Investigator, the sponsor and the Ethics Committee. All such changes must be attached to, or incorporated into, the final protocol, and communicated to all relevant members of Q-Pharm staff and, if appropriate, to trial participants.

- **Non-substantial amendment**

  Administrative or logistical minor changes require a non-substantial amendment. Such changes include but are not limited to changes in study staff or contact details (e.g., Sponsor instead of CRO monitors) or minor changes in the packaging or labeling of study drug. An amendment deemed to be non-substantial must have no ethical implications.

  The implementation of a non-substantial amendment may be done without notification to the HREC. It does not require their approval or to be signed by the investigator.

- **Substantial amendment**

  Significant changes require a substantial amendment. Significant changes include but are not limited to: new data affecting the safety of subjects, change of the objectives/endpoints of the study, eligibility criteria, dose regimen, study assessments/procedures, treatment or study duration, with or without the need to modify the Participant Information Leaflet and Informed Consent.

  Substantial amendments are to be approved by the HREC. The implementation of a substantial amendment can only occur after formal approval by the HREC and must be signed by the investigator.

- **Urgent amendment**

  An urgent amendment might become necessary to preserve the safety of the volunteers included in the study. The requirements for approval should in no way prevent any immediate action being taken by the investigators or the sponsor in the best interests of the subjects. Therefore, if deemed necessary, an investigator can implement an immediate change to the protocol for safety reasons. This means that, exceptionally, the implementation of urgent amendments will occur before submission to and approval by the HREC.

In such cases, the investigator must notify the sponsor within 24 hours. A related substantial amendment will be written within 10 working days and submitted to the HREC, together with a description of the steps which have already been taken in regard to implementation of this amendment.

10.3 Clinical Data Recording

Each volunteer will have a Clinical File (source data) and a Case Report Form (CRF, for protocol specific data) into which relevant data will be recorded.

All recording will be done only in black ink.

Corrections will only be made by drawing a single line through the incorrect entry, writing the correction in the nearest practicable space and initialling and dating the correction. A log of names, signatures and initials of all staff entering data into a Subject's Clinic File and CRF will be kept. Any corrections made after the review and signature of the Principal Investigator will be noted with the initials of the person making the change and countersigned by the Principal Investigator. Correction fluids are not allowed.

All calculations and data transcriptions will be checked by at least one other person, acknowledged by their initials and date.

All deviations from this study protocol will be recorded in the Subject's CRF and included in the final study report. An assessment of the significance of each protocol deviation will be given in the study report.

All CRFs will be reviewed internally by Q-Pharm at the completion of each study visit for any omissions or apparent errors so that these can be corrected without delay.

10.4 Record Retention

All source data, clinical records and laboratory data relating to the study will be retained in the archive of Q-Pharm for 15 years after the completion of the study. All data will be available for retrospective review or audit by arrangement with the Chief Executive Officer of Q-Pharm.

10.5 Biological Samples

Biological samples will be retained for the time required for assessment for analysis, and may then be discarded. Safety serum samples are held with the permission of the subjects for any retrospective safety assessments. An optional Substudy with a separate Participation Information Leaflet and Informed Consent will be used to determine whether samples will be collected for other uses.

10.6 Monitoring

It will be the sponsor's responsibility to ensure that the study is monitored in accordance with the requirements of GCP. The conduct of the study will be reviewed internally by Q-Pharm in accordance with Q-Pharm standard procedures and work instructions and the GCP guidelines.

10.7 Reporting and communication of results

Q-Pharm will provide a summary safety report at the conclusion of the study with all tables and listings as appendices if required.

10.8 Discontinuation of the study

The study sponsor and/or investigators reserve the right to discontinue the study at any time for safety or other reasons. The reasons for this decision will be provided.

After such a decision, the investigators must call in all participating volunteers within a reasonable time period. At this visit all Medical Files and CRFs must be completed as far as possible.

10.9 Study audit

Audits may be carried out by sponsor quality assurance, local authorities or authorities to whom information on this study has been submitted. All documents pertinent to this study must be made available for such inspection after adequate notice of intention to audit.

11 REFERENCES

1. Breman J, Mills A, Snow R, al e. Conquering Malaria. Disease Control Priorities inDeveloping Countries. New York: Oxford University Press; 2006. p. 413-32.
2. Good MF, Stanisic D, Xu H, Elliott S, Wykes M. The immunological challenge to developing a vaccine to the blood stages of malaria parasites. Immunol Rev. 2004 Oct;201:254-67.
3. Marsh K, Kinyanjui S. Immune effector mechanisms in malaria. Parasite Immunol. 2006;28:51-60.
4. Riley EM, Wahl S, Perkins DJ, Schofield L. Regulating immunity to malaria. Parasite Immunol. 2006;28:35-49.
5. Pombo DJ, Lawrence G, Hirunpetcharat C, Rzepczyk C, Bryden M, Cloonan N, et al. Immunity to malaria after administration of ultra-low doses of red cells infected with Plasmodium falciparum. Lancet. 2002 Aug 24;360(9333):610-7.
6. Chatterjee S, Perignon JL, Van Marck E, Druilhe P. How reliable are models for malaria vaccine development? Lessons from irradiated sporozoite immunizations. J Postgrad Med. 2006 Oct-Dec;52(4):321-4.
7. Bejon P, Andrews L, Andersen RF, Dunachie S, Webster D, Walther M, et al. Calculation of liver-to-blood inocula, parasite growth rates, and preerythrocytic vaccine efficacy, from serial quantitative polymerase chain reaction studies of volunteers challenged with malaria sporozoites. J Infect Dis. 2005 Feb 15;191(4):619-26.
8. Cheng Q, Lawrence G, Reed C, Stowers A, Ranford-Cartwright L, Creasey A, et al. Measurement of Plasmodium falciparum growth rates in vivo: a test of malaria vaccines. Am J Trop Med Hyg. 1997 Oct;57(4):495-500.
9. Lawrence G, Cheng QQ, Reed C, Taylor D, Stowers A, Cloonan N, et al. Effect of vaccination with 3 recombinant asexual-stage malaria antigens on initial growth rates of Plasmodium falciparum in non-immune volunteers. Vaccine. 2000 Mar 17;18(18):1925-31.
10. Sanderson F, Andrews L, Douglas AD, Hunt-Cooke A, Bejon P, Hill AV. Blood-stage challenge for malaria vaccine efficacy trials: a pilot study with discussion of safety and potential value. Am J Trop Med Hyg. 2008 Jun;78(6):878-83.
11. Omari AA, Gamble C, Garner P. Artemether-lumefantrine for uncomplicated malaria: a systematic review. Trop Med Int Health. 2004 Feb;9(2):192-9.
12. Andrews L, Andersen RF, Webster D, Dunachie S, Walther RM, Bejon P, et al. Quantitative real-time polymerase chain reaction for malaria diagnosis and its use in malaria vaccine clinical trials. Am J Trop Med Hyg. 2005 Jul;73(1):191-8.
13. Rao M. Scalable human ES culture for therapeutic use: propagation, differentiation, genetic modification and regulatory issues. Gene Ther. 2008 Jan;15(2):82-8.
14. Solves P, Mirabet V, Perales A, Carbonell-Uberos F, Roig R. Banking strategies for

improving the hematopoietic stem cell content of umbilical cord blood units for transplantation. Curr Stem Cell Res Ther. 2008 May;3(2):79-84.

15. Gaziano TA, Young CR, Fitzmaurice G, Artwood S and Gaziano JM. Laboratory based versus non-laboratory method for assessment of cardiovascular disease risk: the NHANES I Follow-up Study cohort. Lancet. 2008 March 15; 371:923-31.

12 SIGNED AGREEMENT TO STUDY PROTOCOL

See next page.

A PHASE II STUDY OF THE EFFICACY OF ANTIMALARIAL DRUGS AGAINST *PLASMODIUM FALCIPARUM* BY EXPERIMENTAL CHALLENGE WITH A LOW DOSE OF BLOOD STAGE PARASITES IN HEALTHY VOLUNTEERS

(Protocol Number: QP09C08)

This protocol has been read and approved by the undersigned Principal Investigator conducting the study at Q-Pharm Pty Limited who acknowledges that:

1. They assume responsibility for the proper conduct of the study at this site.
2. They will conduct the study in compliance with this protocol, any mutually agreed future protocol amendments, and with any other study conduct procedures provided by the sponsor.
3. They will not to implement any changes to the protocol without agreement from the sponsors and prior review and written approval from the relevant study ethical review committees, except where necessary to eliminate an immediate hazard to the participants, or for administrative aspects of the study (where permitted by all applicable regulatory requirements).
4. They will be thoroughly familiar with the appropriate use of the drug, as described in this protocol, and any other information provided by the sponsors, including, but not limited to,: the current Investigator's Brochure (IB) and any IB supplement (if applicable).
5. They will comply with, ICH and current "Good Clinical Practices" (GCP), the Declaration of Helsinki and the NHMRC National Statement on Ethical Conduct in Research Involving Humans and all applicable regulatory requirements.
6. They will accept the oversight of appointed monitor(s) and control procedures.

______________________________ Date: __________________

James S. McCarthy MBBS
Principal Investigator

This protocol has been read and approved by the study sponsors:

______________________________ Date: __________________

____

Stephan Duparc
MMV Medical Director
on behalf of Medicines for Malaria Venture
