# Supplementary material for: A Pilot Randomised Trial of Induced Blood-Stage Plasmodium falciparum Infections in Healthy Volunteers for Testing Efficacy of New Antimalarial Drugs
Source: PLoS One. 2011 Aug 22;6(8):e21914. doi: 10.1371/journal.pone.0021914 (PMC3159571; doi:10.1371/journal.pone.0021914)
Supplement: Protocol Amendment S1 — Amended trial protocol. (PDF) [file pone.0021914.s005.pdf]

15March 2010

Re: P1286 A PILOT PHASE II STUDY OF THE EFFICACY OF ANTIMALARIAL DRUGS AGAINST PLASMODIUM FALCIPARUM BY EXPERIMENTAL CHALLENGE WITH A LOW DOSE OF BLOOD STAGE PARASITES IN HEALTHY VOLUNTEERS (Protocol No. QP09C08)

RE: Expedited Approval post Cohort 1 and Review of protocol by Global Safety Board of Medicines for Malaria Venture for remainder of study

Dear Ian,

Thank you for taking my calls and emails in advance to keep you up to date with this clinical trial and its ongoing activity. Following up from the advanced email outlining possible changes, these have now been reviewed globally and final details are described below. We are aiming at dosing Cohort 2 on Wednesday 17Mar2010 with the new protocol and ICF if possible.

As was outlined in the previously circulated Protocol Deviation Report, during the study QP09C08 an error was made in preparation of the inoculum (See Protocol Deviation_Cohort 1_innoculum). According to the methods laid out in the approved protocol, an estimated dose of 5300 parasitised RBC, of which 1,800 were estimated to be viable was the dose to be administered. The calculated inoculum that was actually administered was about 100 parasitised RBC (with an initial estimate of 50% viability). That a smaller dose was administered is in accordance with the PCR data that accrued during the study, namely that onset of detectable parasitemia (by PCR) was two days later than was expected, and the target parasitemia intended to be reached by evening Day 6 was not reached. These data are also consistent with the reported parasite growth following inoculation of about 30 viable parasitised RBC. (Pombo et al.) The possibility of delaying treatment was discussed internally but judged inappropriate.

Following the protocol deviation two actions were taken:

1. An internal investigation to identify the cause and to develop a strategy to reduce the risk for this error to recur. This has entailed rewriting the working instructions for preparation of the inoculum, and consideration of omitting the haemocytometer count (see below).

2. An investigation of alternate procedures to quantify the size of the inoculum, both the number of parasites and red cells infused. Investigations were undertaken on both residual aliquots of the actually injected inoculum and on a second aliquot that was thawed and processed in an identical fashion. Evaluation included Flow cytometry using three different methods, qPCR, and assessment of percent haemolysis.

It should be stated that he calculations used to determine the size of the inoculum dose were based on assumptions that $212p/5x10^6$ RBC cells are present in the stock inoculum. This assumption had been used by all past investigators using this inoculum to calculate the dose using an identical formula. However, flow cytometric analyses indicated that the “assumed” parasite density in the stock inoculum is unlikely to be accurate. Possible reasons for this include significant haemolysis at the time of thawing, potentially differential haemolysis of infected and uninfected red cells, and deterioration of the stored vials (loss of “potency”) over 18 years of storage.

With data to hand it is the view of the investigators, and of parties who have provided expert external advice that at this stage the best measure of dose and viability is parasite growth *in vivo*, and that the best approach to improving reproducibility of the study methodology is to prepare the aliquot in a standard way, dose an agreed volume but one not exceeding one more than what is considered prudent by the team and advisors, and to closely monitor volunteers for the onset of submicroscopic parasitemia (by PCR) and reaching the target parasitemia.

The progress of the trial, specifically the safety issues and inoculum preparation were reviewed in a scheduled Safety Review Team teleconference on Friday March 15, 2010. It was concluded that NO safety issues had arisen in cohort 1 and that cohort 2 could proceed on the basis of this. However a number of significant amendments were agreed to, subject to them receiving approval from the HREC. These were all aimed at ensuring a study endpoint could be reached while still maintaining volunteer safety. In brief the amendments entail:

1. Simplification of the inoculum preparation to reduce potential for error by:
    a. Removal of the microscopy step to quantify red cells by haemocytometer chamber counting
    b. Instead inoculate 250 µL cell suspension in 2 mL (a total of~ 5300 pRBC)
2. Delay admission for inpatient observation if volunteer(s) not positive PCR by Day 5
3. Administer antimalarial drug therapy based on PCR parasitaemia (≥ 1,000 parasites per mL) rather than on a pre-specified day and time. This will enable delay of treatment if parasite growth is slower than anticipated, or earlier if faster.
4. Reduce cohort 2 size to 4 volunteers to enable iterative changes to the protocol to improve probability of reaching study outcome.
5. At the discretion of the Clinical Investigator to allow participants to go home 36 hours after commencing therapy, but only if completely asymptomatic and tolerating antimalarial therapy. In this circumstance volunteers would continue to be followed on an outpatient basis twice daily as per protocol and continue to receive designated antimalarial drug treatment under direct observation whilst attending the Q-Pharm unit on scheduled twice daily visits.
6. An additional interim review of cohort 2 data has been agreed to before making a decision further cohort(s).

Please find attached a modified Protocol, Schedule of events and Consent form.

We look forward to your review of the protocol. Subject to it being judged satisfactory we plan to begin this cohort on Wednesday March 17.

If you have any questions or are seeking any clarification, please feel free to call me on my mobile (0414424659) to discuss.

Sincerely

Dr James McCarthy

Main Changes made **ONLY to the Appendix 4** – PICF AND **Appendix 6** – Preparation of malaria inoculum to reflect the need to address the Protocol Deviation and review of the data from Cohort 1

Appendices have been updated in version and separated for convenience.

NO changes were made to Appendix 3, 5, 7, 8, 9

Update documents for Expedited Approval 15Mar 2010 :

- 2010-03-15 18:29:45 Appendix 4 PICF_V3_15 March2010_Tracked
- 2010-03-15 18:20:49 Appendix 4 Pariticipant Information Sheet_V3_15 March2010
- 2010-03-15 18:20:31 Appendix 8_9_10 AE tables_V3_15 March2010
- 2010-03-15 18:18:44 Appendix 7 CMI and PI of Malaria Drugs_V3_15 March2010
- 2010-03-15 18:17:34 Appendix 6 Preparation of Malaria Inoculation_V3_15 March2010
- 2010-03-15 18:17:17 Appendix 5 Symptoms and Signs_V3_15 March2010
- 2010-03-15 18:16:57 Appendix 3 Med History and PE_V3_15 March2010
- 2010-03-15 18:16:44 Appendix 2 Lab Evaluations_V3_15 March2010
- 2010-03-15 18:16:32 Appendix 1 Schedule of Events_V3_15 March2010
- 2010-03-15 18:16:17 APPENDICES QP09C08 Listings
- 2010-03-15 18:16:01 QP09C08_MMV protocol_ V3_15Mar2010
- ~~2010-03-15 18:15:40 *--previous version of 'QP09C08_MMV protocol_ V3_15Mar2010'~~
- ~~2010-03-15 18:15:28 *--previous version of 'QP09C08_MMV protocol_ V3_15Mar2010'~~
- ~~2010-03-15 18:14:36 *--previous version of 'QP09C08_MMV protocol_ V3_15Mar2010'~~
- ~~2010-03-15 18:14:26 *--previous version of 'QP09C08_MMV protocol_ V3_15Mar2010'~~
- 2010-03-15 14:54:27 QP09C08_ Protocol Deviation_Cohort 1_Innoculum

The invoice for this project is to be submitted to:

Natasha Hayes
Clinical Research Associate

Trident Clinical Research Pty Ltd.
Suite 37, 2 Benson St, Toowong, QLD 4066
Ph: +61 (0)7 3217 7711
Fax: +61 (0)7 3217 7511
Mobile:+61 (0)430 215 563
mail: nhayes@tridentclinicalresearch.com
www.tridentclinicalresearch.com
